# Quantitative Proteomic Analysis on the Slightly Acidic Electrolyzed Water Triggered Viable but Non-Culturable *Listeria monocytogenes*

**DOI:** 10.3390/ijms241310616

**Published:** 2023-06-25

**Authors:** Hsin-Yi Chang, Chin-Ying Gui, Tsui-Chin Huang, Yen-Con Hung, Tai-Yuan Chen

**Affiliations:** 1 Graduate Institute of Medical Sciences, National Defense Medical Center, Taipei 11490, Taiwan; hsinyi.chang@mail.ndmctsgh.edu.tw; 2 Department of Research and Development, National Defense Medical Center, Taipei 11490, Taiwan; 3 Department of Food Science, National Taiwan Ocean University, Keelung 20224, Taiwan; 00539055@email.ntou.edu.tw; 4 Graduate Institute of Cancer Biology and Drug Discovery, College of Medical Science and Technology, Taipei Medical University, Taipei 11031, Taiwan; tsuichin@tmu.edu.tw; 5 Department of Food Science & Technology, University of Georgia, Griffin, GA 30223-1797, USA; yhung@uga.edu

**Keywords:** *Listeria monocytogenes*, slightly acidic electrolyzed water, viable but non-culturable state, quantitative proteomics, chlorinated protein

## Abstract

This study undertakes a comprehensive exploration of the impact of slightly acidic electrolyzed water (SAEW) on *Listeria monocytogenes*, a common foodborne pathogen, with a particular focus on understanding the molecular mechanisms leading to the viable but nonculturable (VBNC) state. Given the widespread application of SAEW as an effective disinfectant in the food industry, uncovering these molecular pathways is crucial for improving food safety measures. We employed tandem mass tags (TMT), labeling proteomic techniques and LC-MS/MS to identify differentially expressed proteins under two doses of SAEW conditions. We indicated 203 differential expressed proteins (DEPs), including 78 up-regulated and 125 down-regulated DEPs. The functional enrichment analysis of these proteins indicated that ribosomes, biosynthesis of secondary metabolites, and aminoacyl-tRNA biosynthesis were enriched functions affected by SAEW. Further, we delved into the role of protein chlorination, a potential consequence of reactive chlorine species generated during the SAEW production process, by identifying 31 chlorinated peptides from 22 proteins, with a dominant sequence motif of Rxxxxx[cY] and functionally enriched in translation. Our findings suggest that SAEW might prompt alterations in the protein translation process and trigger compensatory ribosome biosynthesis. However, an imbalance in the levels of elongation factors and AARSs could hinder recovery, leading to the VBNC state. This research carries substantial implications for food safety and sanitation, as it adds to our understanding of the SAEW-induced VBNC state in *L. monocytogenes* and offers potential strategies for its control.

## 1. Introduction

*Listeria monocytogenes* is a Gram-positive, rod shape and non-spore forming bacterium. The Foodborne Disease Active Surveillance Network (FoodNet) reported that 104 listeriosis cases, leading to 22 deaths and a 95% hospitalization rate within the surveillance populations in 2020 [1]. *L. monocytogenes* is widely found in moist environments and can further transmit to food, especially to ready-to-eat (RTE) foods such as fresh produce, unpasteurized milks, soft cheese, RTE meats, raw or smoked fish and other seafood [2]. It has been reported that *L. monocytogenes* can survive and enter a viable but non-culturable state (VBNC) under various stresses, such as sanitizers, pH, and process temperatures. VBNC bacteria cannot be detected using the conventional plate count method but still retains metabolic activity which may revert to the culturable state through resuscitation. Regardless of whether the pathogenic bacteria is in VBNC or in a resuscitated state, they retain the ability to express virulence genes and hence pose a potential health risk to human beings [3,4].

Electrolyzed water (EW) is generated through the electrolysis of sodium chloride (NaCl) or diluted hydrochloric acid (HCl) solutions in a specialized chamber, which may or may not contain a diaphragm. The electrolysis process can yield different types of EW, including slightly acidic electrolyzed water (SAEW). SAEW has a mild pH, typically around 5–6.5, and retains the potent disinfecting properties of EW while being gentler on surfaces, making it particularly suitable for applications in the food industry. EW and SAEW represent significant technological advancements with versatile applications. Their unique properties, encompassing broad-spectrum activity, low toxicity, environmental compatibility, user-friendliness, and usability, make these solutions effective disinfectants and preferred choices in ensuring food safety and sanitation [2,5,6].

Despite the success of EW as a disinfectant, there remain unanswered questions regarding its interaction with bacteria, particularly the formation mechanism of viable but nonculturable (VBNC) bacteria under EW stress. VBNC bacteria are a unique state of bacteria where they are alive and can still cause infection but cannot be cultured on standard laboratory media [7,8,9]. Understanding the transition mechanism to this state under the influence of EW could provide significant insight into bacterial resistance and survival strategies.

Proteomics, the study of proteins at a global scale, is a relatively new but rapidly evolving field. It encompasses the comprehensive analysis of proteins, including their identities, abundances, functions, structures, interactions, networks, and post-translational modifications. These collective insights allow us to better understand the cellular functions and the complex molecular mechanisms underlying physiological and pathological states [10,11]. Quantitative proteomics, in particular, has revolutionized the field by providing qualitative and quantitative information about the proteome. It employs several isotope labeling techniques, enabling the measurement of changes in protein abundance under different conditions. These techniques include stable isotope labeling with amino acids in cell culture (SILAC), isobaric tags for relative and absolute quantification (iTRAQ), and tandem mass tags (TMT), among others [12,13,14]. SILAC allows for the incorporation of isotopically labeled amino acids during protein synthesis, while iTRAQ and TMT employ chemical labels that are attached to the peptides after protein digestion. These labels are identical in chemical structure but differ in isotopic composition, enabling differentiation and quantification in a mass spectrometer. The isotope labels in these techniques serve as ‘mass tags’ that can be detected and quantified, providing comparative data on protein expression levels between different samples. Differential proteomics, facilitated using these quantitative methodologies, has been broadly utilized in deciphering the alterations in the proteome in response to different physiological or pathological conditions. The data generated through these quantitative proteomics techniques provide valuable insights into the dynamic nature of the proteome, informing our understanding of how cells adapt and respond to external stimuli or stressors [12,13,14].

This study aims to identify the concentration thresholds of SAEW that induce a VBNC state in *Listeria monocytogenes*, and to elucidate the underlying molecular mechanism. Upon confirming the VBNC state using traditional plating methods, scanning electron microscopy, and flow cytometry, we apply TMT-labeling quantitative proteomic analysis to explore the proteomic alterations during the VBNC transition. Furthermore, we have identified that several chlorination sites were highly elevated by SAEW. We further performed functional enrichment analysis using Gene Ontology (GO) terms and Kyoto Encyclopedia of Genes and Genomes (KEGG) pathways, and constructed protein-protein interaction networks using STRING. Ultimately, we provide comprehensive insights into the formation mechanism of SAEW-induced VBNC bacteria.

## 2. Results

### 2.1. The Culturability of L. monocytogenes after Treatment with Slightly Acidic Electrolyzed Water (SAEW)

To estimate the required concentration for producing VBNC *L. monocytogenes*, we treated the cells with SAEW for 5 min at a range of concentration. For SAEW at 4 and 6 mg/L of available chlorine concentrations (ACCs), only 0.31 and 0.72 log CFU/mL reductions were achieved, while *L. monocytogenes* could be completely inactivated when treated with high concentration of SAEW (>8 mg/L ACC), indicating that these concentrations of chlorine lead the cells in a nonculturable state (Table 1).

### 2.2. Determination of Cell Viability of L. monocytogenes Using Flow Cytometry

**Figure 1:**
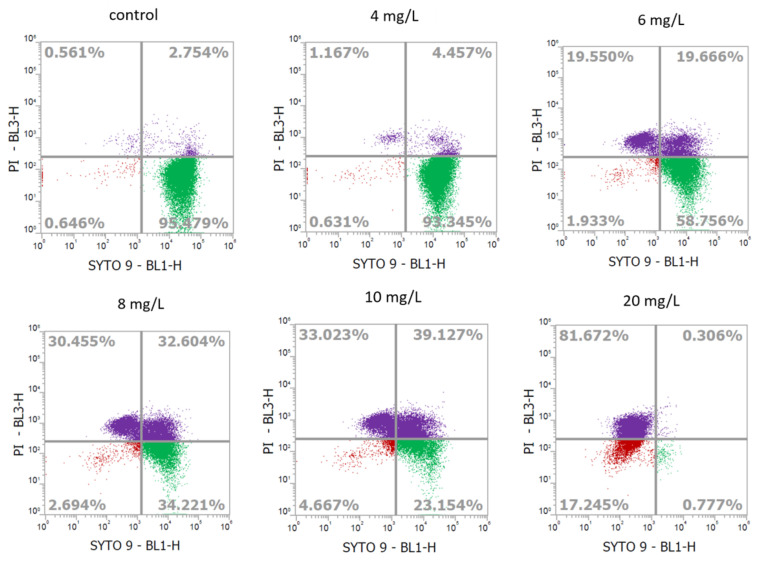
Flow cytometry profiles showing the fate of *L. monocytogenes* (BCRC14845) cells after treatment with different concentrations of slightly acidic electrolyzed water. *X*-axis: Green fluorescence collected at 530 nm; *Y*-axis: Red fluorescence collected at 695 nm.

To determine the viability of SAEW-treated cells, we applied the LIVE/DEAD^TM^ BacLight^TM^ bacterial viability kit, composed of two fluorescent dyes SYTO9 and propidium iodide (PI). SYTO^®^ 9 can penetrate all cells and produces green fluorescence while PI only penetrates membrane damaged cells and shows red fluorescence. *L. monocytogenes* that did not form colonies on plates but exhibited more than 20% cell population in the viable region of the flow cytogram were defined as VBNC cells. When treated with 20 mg/L of ACC for 5 min, approximately 80% of cells located in the dead region, and nearly no cells were in the viable region, suggesting that these cells were neither viable (Figure 1) nor culturable (Table 1). The population shifted to the viable region with decreasing concentration of ACCs. About 23 to 34% of the cells were located in a viable region with under 8 and 10 mg/L of ACC treatments while standard plating results showed ND in both treatments, indicating that treatment of ACCs in 8 and 10 mg/L could induce VBNC cells.

### 2.3. Morphological Changes of VBNC L. monocytogenes Using Scanning Electron Microscopy

**Figure 2:**
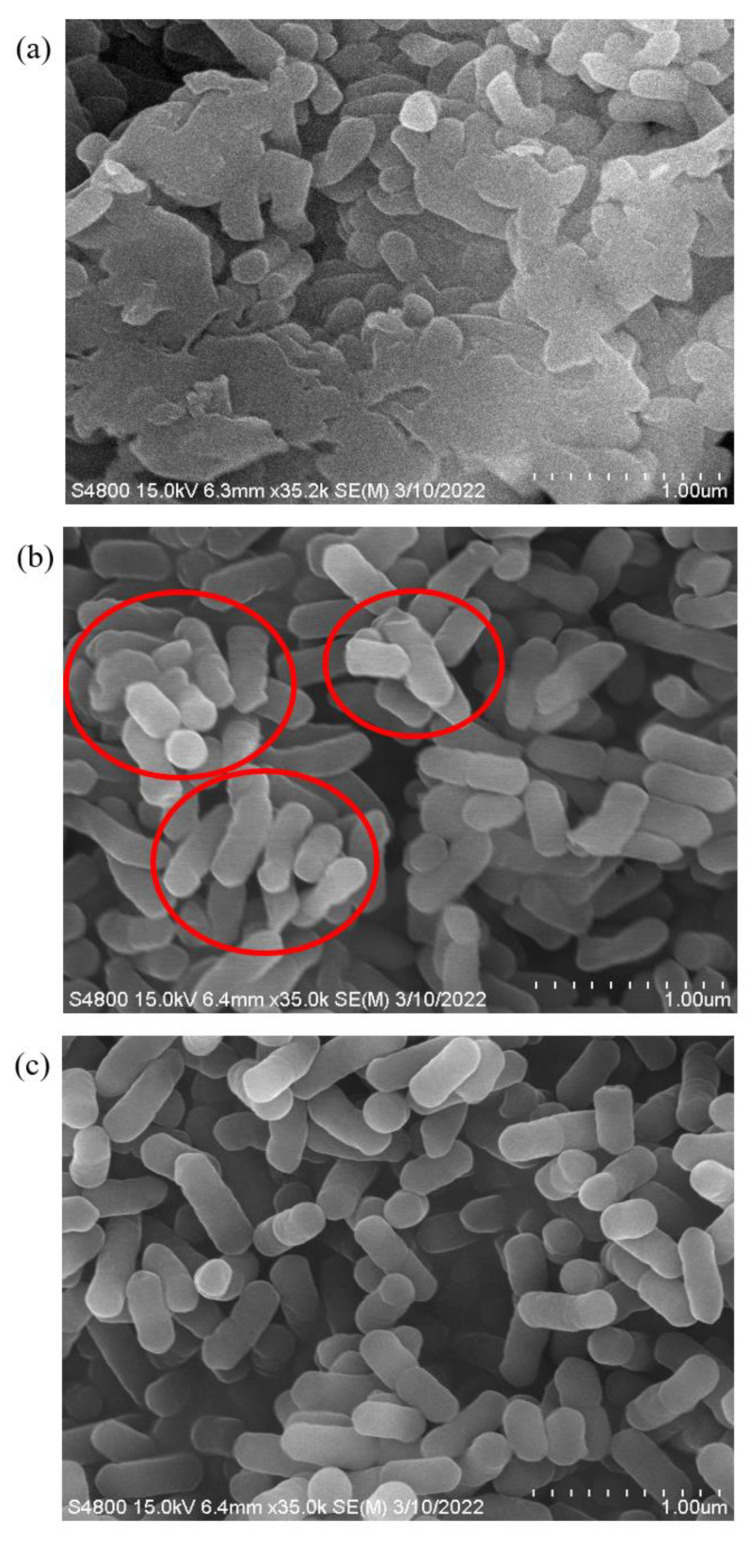
Morphological characteristics of *L. monocytogenes* under scanning electron microscopy (Magnification 35,000×). SEM (**a**) dead cells treated with 20 mg/L of ACC in SAEW, and (**b**) VBNC cells treated with 10 mg/L of ACC in SAEW, (**c**) live cells treated with DI water as control.

To monitor the morphological changes of *L. monocytogenes* at different states, we collected cells treated with three conditions, DI water (CTL), 10 mg/L ACC (SA10), and 20 mg/L ACC (SA20) to represent live cells, VBNC cells, and dead cells. The cells treated under SA20 showed significant cell membrane damage and cell aggregation (Figure 2a). On the other hand, the SA10 treatment, which prompted the cells to enter the VBNC state, did not cause membrane damage; the majority of the cells remained almost intact, as demonstrated in Figure 2b. Comparing with the control cells (Figure 2c), we noticed a shrinkage of the cell membrane (indicated with the red circle in Figure 2b) and the formation of aggregations in the VBNC cells.

### 2.4. Differential Expressed Proteins (DEPs) of L. monocytogenes after Treating with SAEW

**Figure 3:**
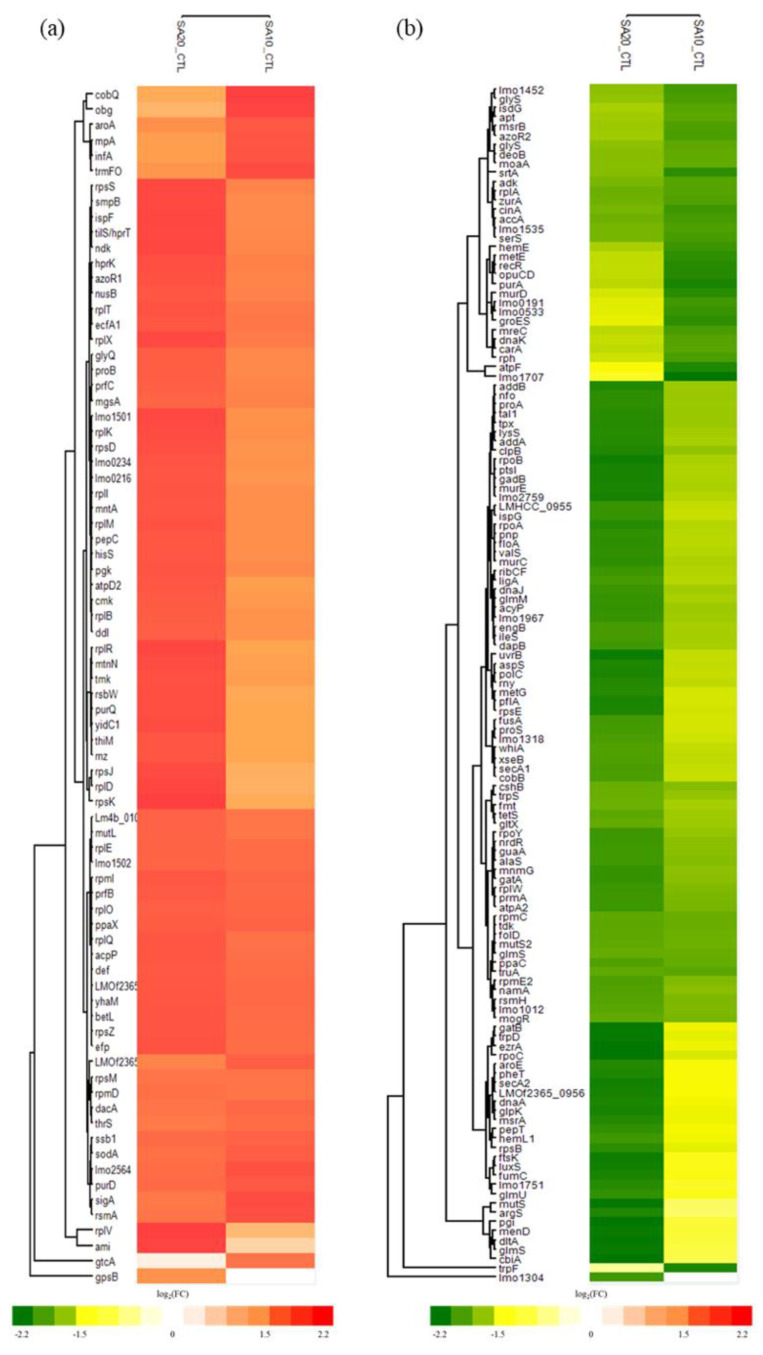
Cluster analysis of (**a**) up-regulated and (**b**) down-regulated DEPs in *L. monocytogenes* under different concentration of SAEW.

In a comprehensive effort to investigate the molecular mechanism behind the induction of the VBNC state in *L. monocytogenes* using slightly acidic electrolyzed water (SAEW), we utilized quantitative proteomics in this study. Specifically, we applied tandem mass tags (TMT) labeling to decipher global proteome changes in *L. monocytogenes* under various conditions: Deionized water (CTL), 10 mg/L ACC (SA10), and 20 mg/L ACC (SA20). In total, 534 proteins were identified, 203 proteins with *p* < 0.05 in the comparison with the control group were considered as differentially expressed proteins (DEPs). Of the 203 proteins, 78 proteins were up-regulated and 125 down-regulated (Figure 3). Considering the proteins within the 203 DEPs that exhibited fold changes of ≥±1, we considered these as significant DEPs (sDEPs). This subset consisted of 16 proteins, including 11 that were up-regulated (Table S1, Figure S1) and 5 that were down-regulated (Table S2). The significantly up-regulated proteins (rpsZ, rpmI, lmo1306, betL, lmo2564, rplX, rpmD, rplT, rplO, rnpA, and lmo0208) were found to be predominantly associated with the ribosomal function (*p*-value = $1.8\times 10^{-6}$, Benjamini adjusted *p*-value = $1.5\times 10^{-5}$). Conversely, the significantly down-regulated proteins (lmo1608, floA, trpD, gadB, and glyS) were primarily cytosolic proteins (Table S2).

### 2.5. GO and KEGG Functional Enrichment Analysis of SAEW-Regulated Proteins

**Figure 4:**
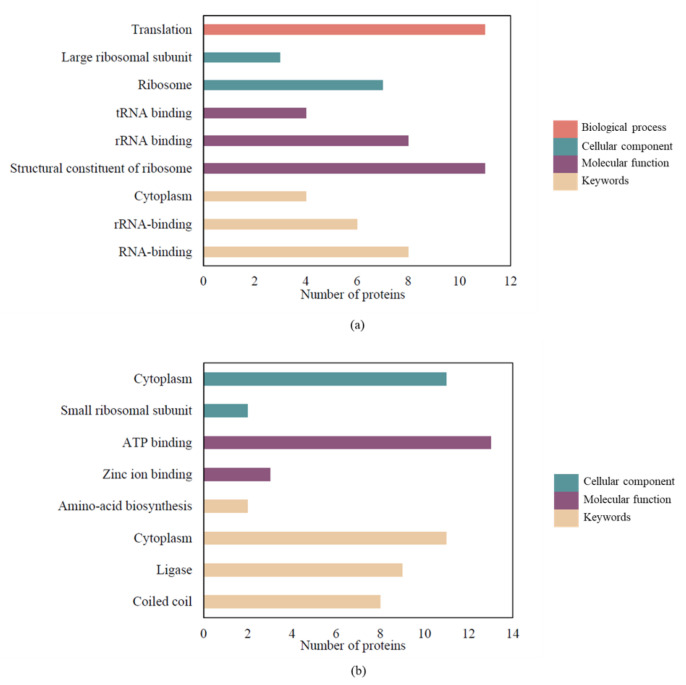
Histogram of GO and Keywords enrichment of (**a**) up-regulated and (**b**) down-regulated proteins. The functional annotation of DEPs was performed using DAVID (v. 6.8).

To further understand the effects of SAEW on *L. monocytogenes*, we performed functional enrichment analysis on DEPs (full results are listed in Table S3). Functional enrichment analysis on ‘omics’ data provides a valuable biological context to large lists of differentially expressed genes or proteins by grouping them into functionally related categories. This process helps simplify complex data, promotes the generation of new research hypotheses, and enables the comparison of different experimental conditions. The up-regulated proteins were primarily associated with the biological processes of translation, as well as the cellular component and molecular function related to the ribosome, according to Gene Ontology (GO) terms. RNA-binding emerged as the most enriched term among the UniProtKB Keywords for up-regulated proteins. Conversely, for the down-regulated proteins, GO analysis highlighted significant enrichment in the cellular component of the cytoplasm and the molecular function of ATP binding (Figure 4). Pathway analysis using the Kyoto Encyclopedia of Genes and Genomes (KEGG) revealed that a majority of these pathways were related to metabolic processes, the ribosome, the biosynthesis of secondary metabolites, and aminoacyl-tRNA biosynthesis in the VBNC state. The metabolic pathway comprised 45 down-regulated and 17 up-regulated proteins (Table S3). Twenty up-regulated and six down-regulated DEPs were related to the ribosome. These results align with our GO analysis (Figure 4) wherein up-regulated proteins prominently featured in the ribosome (cellular component) and structural components of the ribosome (molecular function), while the down-regulated proteins were more related to aminoacyl-tRNA biosynthesis.

### 2.6. SAEW-Mediated Protein Chlorination in VBNC L. monocytogenes

**Figure 5:**
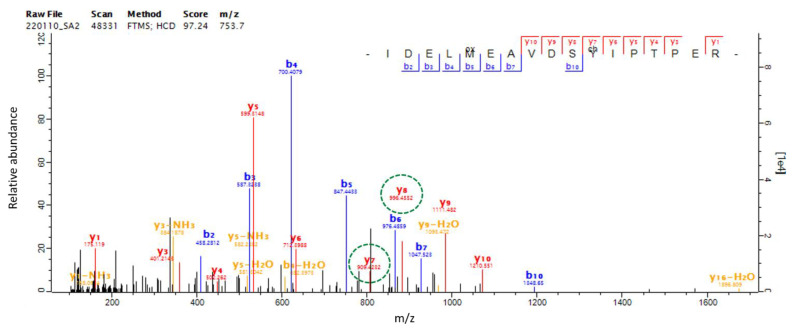
The mass spectrum of higher energy collision dissociation (HCD) of the chlorinated peptide at *m/z* 753.7. The spectrum was matched to the sequence IDELMEAVDS^199^Y^Cl^IPTPER of protein tuf.

**Figure 6:**
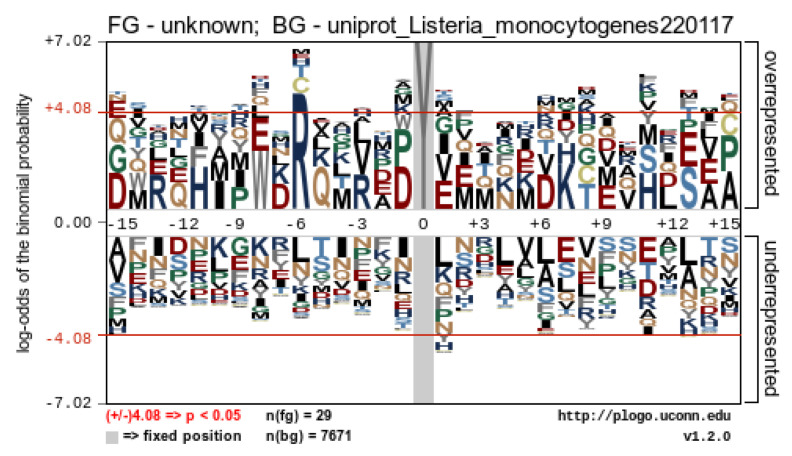
The sequence motif analysis of chlorinated protein in *L. monocytogenes*. The image was generated in pLogo. The red horizontal lines suggest a threshold of *p* < 0.05.

The production of SAEW through the electrolysis process produces various reactive species, including chlorine, known for its strong oxidizing capabilities. This chlorine has the potential to covalently bind to proteins through a process known as chlorination, thereby modifying their structure. To understand the extent and specifics of this modification, we further investigated whether SAEW induced chlorination and, if so, which specific proteins were impacted and at which residues. By applying chlorination on tyrosine (Y) and tryptophan (W) as variable modifications during peptide searching, we identified many chlorinated peptides which could be further characterized using high energy collision dissociation (HCD)-derived MS/MS spectra. In total, 31 chlorinated peptides from 22 proteins were discovered, with 26 chlorinated Y peptides, 3 chlorinated W, and 2 chlorinated Y and W peptides (Table 2). Most of the chlorinated proteins were upregulated and showed chlorine concentration dependently. The most abundant chlorinated protein was elongation factor Tu (tuf), comprising six peptides containing tyrosine chlorination at Y41, Y88, Y130, Y161, Y199, and Y269 (Table 2). For instance, a representative peptide IDELMEAVDS^199^Y^Cl^IPTPER at *m*/*z* 753.7 derived from the EF-Tu protein has been identified with the chlorinated position at Y199 shown (Figure 5). The difference of the *m*/*z* between the y_7_ ion at *m*/*z* 909.428 and the y_6_ ion at *m*/*z* 712.899 was 196.529 *m*/*z*, indicating a chloro-tyrosine moiety. The HCD MS/MS spectra of the other five chlorinated peptides from the EF-Tu protein are shown in Supplementary Figures S2–S6. We next analyzed whether the chlorinated peptides exhibited a consensus sequence motif near the chlorinated residues using pLogo and revealed a significant overrepresentation of arginine (R) at position −6 (Figure 6). The six chlorinated sites including Y112 of pcrB in Y112, Y201 of groEL, Y61 of rpsS, and Y45, Y130, and Y161 of tuf, exhibited the featured motif Rxxxxx[cY] (Table 2, Figure 6). To interpret the possible effects of chlorination based on the structural alteration, we constructed their protein-protein interaction network using STRING and performed functional enrichment analysis using the GO database. The chlorinated proteins were highly connected (PPI enrichment *p*-value = $7.23\times 10^{-6}$) and functionally enriched in chaperone cofactor-dependent protein refolding, translation, and organophosphate metabolic process (Figure S7).

## 3. Discussion

Foodborne pathogens, entering the viable but nonculturable (VBNC) state during various stages of food handling, such as preparation, processing, preservation, storage, and distribution, pose significant risks to food safety and have been thoroughly examined in past literature [3,4]. These VBNC or injured bacteria could potentially trigger foodborne illnesses or food spoilage; yet, being unculturable on plates but able to resuscitate back to culturable state may produce false-negative treatment results [7,8,9]. Slightly acidic electrolyzed water (SAEW), a disinfectant widely used in the industry, has been shown to induce the VBNC state in certain bacteria [15,16]. For instance, studies have demonstrated that *E. coli* and *L. monocytogenes* can transition into the VBNC state when exposed to 2.5 and 1.25 mg/L of available chlorine concentration (ACC) in acidic electrolyzed water, respectively [7]. Similarly, chlorine solution has been found to induce the VBNC state in *L. monocytogenes* and *Salmonella enterica* at ACCs of 12 and 3 mg/L, respectively [17]. *L. monocytogenes*, in particular, was found to be completely eliminated using SAEW at an ACC of 12 mg/L [18]. Our findings, involving ACCs of 8–10 mg/L, are consistent with these observations (Table 1 and Figure 1). Differences in these results might arise due to varying types of electrolyzed water used and the ratio of bacteria to SAEW. For instance, a previous study employed a bacteria-to-SAEW ratio of 1:9 [18], while our research utilized a 2:3 ratio. Despite these findings, studies exploring VBNC formation in Gram-positive bacteria induced by SAEW are relatively sparse.

There were studies on some protein biomarkers of VBNC bacteria under various stressors [19,20], the proteomic analysis of VBNC bacteria induced by electrolyzed water has yet to be investigated. Transcriptomic analyses have suggested that VBNC bacteria can modify their primary metabolic pathways to optimize energy acquisition and decrease metabolic rates to survive unfavorable conditions [21,22]. In this study, we examined the impact of SAEW on inducing the VBNC state in *L. monocytogenes* and conducted a comprehensive global protein expression analysis using TMT labeling-LC-MS/MS, aiming to decipher the underlying formation mechanisms of VBNC bacteria.

GO functional enrichment analysis in this study revealed significant enrichment of “structural constituent of ribosome” and “translation” within up-regulated proteins, suggesting that VBNC cells may not be dormant but could actively promote protein synthesis to sustain the metabolic rate amidst environmental stress. Protein synthesis occurs through translation processes on ribosomal subunits, comprising initiation, elongation, and termination stages. During initiation, initiation factors interact with GTP, N-formyl-Met-tRNA^fMet^, mRNA, and a free 30S small subunit to form a 30S initiation complex. Upon addition of the 50S subunit, a 70S initiation complex is created, primed for the elongation stage. We observed the upregulation of many ribosomal proteins and the initiation factor IF-1 (infA) in the KEGG pathway (Tables S1 and S3), suggesting that SAEW treatment promotes the initiation of protein synthesis. In the subsequent elongation stage, EF-Tu (elongation factor Tu) binds to aminoacyl-tRNA and GTP, facilitating the translation process on the 70S ribosome complex. Aminoacyl-tRNA synthetases (AARSs) are a diverse family of enzymes responsible for attaching specific amino acids to their corresponding tRNAs, with at least 20 individual AARSs for each aa-tRNA. GatCAB, an essential heterotrimeric, glutamine-dependent enzyme complex involved in the transamidation of Glu-${\mathrm{tRAN}}^{\mathrm{Gln}}$ and Asp-${\mathrm{tRAN}}^{\mathrm{Asn}}$, was also investigated. GatA and GatB, subunits of GatCAB that play roles in ammonia production and misacylated tRNA activation and transamidation, respectively, exhibited down-regulation, possibly reducing the formation of Glu-${\mathrm{tRAN}}^{\mathrm{Gln}}$ and Asp-${\mathrm{tRAN}}^{\mathrm{Asn}}$ [23,24]. Furthermore, 14 proteins related to aminoacyl-tRNA biosynthesis were down-regulated, indicating that SAEW treatment might impede peptide chain elongation in protein synthesis (Tables S2 and S3). Together with the observed shortage of EF-Tu, AARSs, and translation-related proteins, our findings suggest a down-regulation of the translation process under SAEW treatment.

The low-temperature induced VBNC *E. coli* previously reported a transformation from typical rod shape to shorter rods, and a decrease in cell size, as seen under SEM observation [25]. This morphological change aligns with our findings where VBNC *E. coli* maintained an almost intact membrane following low chlorine concentration treatment [22], suggesting cells might minimize cellular maintenance requirements to enhance survival under environmental stress [26]. Interestingly, the downregulation of murC, murD, and murE (Table S2) suggests decreased cell wall (peptidoglycan) biosynthesis. The Mur enzyme family, which plays a crucial role in peptidoglycan formation, includes transferases (MurA), dehydrogenases (MurB), amino acid ligases (MurC to MurF, and Ddl), racemases (Alr, DadX, and MurI), and glycosyltransferases (MraY, MurG) [27]. In particular, MurC to MurF are ATP-dependent amide bond ligases that sequentially add polypeptide chains to form a component of the peptidoglycan layer, UDP-N-acetylmuramoyl-L-alanine-D-glutamate-meso-diaminopimelate-D-alanyl-D-alanine (UDPMurNAc-pept) [28]. As the cell wall provides structure and maintains bacterial shape, down-regulation of these proteins might lead to changes in bacterial morphology [29], as observed in this study. A proteomic analysis of VBNC soil bacteria similarly reported down-regulation of cell shape-related proteins, which aligns with our findings. In *Cupriavidus metallidurans*, proteins related to cell shape and protein synthesis, including EF-Tu, were down-regulated during the transition to the VBNC state [29]. While reductions in EF-Tu levels have been reported to have minimal effects on translation, they can substantially impact cell shape and MreB localization, thereby modulating cell shape in response to environmental stress [30]. Our observations of cell shrinkage in the VBNC state and down-regulation of mur family proteins provide further evidence of the role of EF-Tu role in influencing cell shape (Figure 2 and Table S2).

Quorum sensing (QS) is a cell density-dependent communication mechanism that uses chemical signals to regulate the expression of genes involved in specific bacterial processes [31]. One such system, the luxS/AI-2 QS system, operates using AI-2 (autoinducer-2) as a signaling molecule and is found in both Gram-negative and Gram-positive bacteria. It modulates a range of cellular processes, including the production of virulence factors, luminescence, sporulation, motility, toxins, biofilm formation, and drug resistance [32]. LuxS, a pivotal enzyme in this system, generates AI-2 through the catalysis of S-ribosylhomocysteine (SRH) and also plays a critical role in the activated methyl cycle, which is integral to bacterial metabolism. Quorum sensing inhibition can potentially impede biofilm formation [33]. Intriguingly, a down-regulation of the luxS gene has been observed in the VBNC state of *Proteus mirabilis*, potentially impacting its biofilm formation capacity [34]. Similarly, our study noted a down-regulation of luxS in VBNC *L. monocytogenes* (Tables S2 and S3), which might result in the inactivation of quorum sensing and inhibition of biofilm formation.

SAEW contains hypochlorous acid (HOCl) as its primary active component, which has been shown to cause extensive oxidative modification of proteins, resulting in irreversible tissue damage and cell death [35]. Chlorination stoichiometry at a specific site in a protein refers to the ratio of the total amount of protein chlorinated at that site to the total amount of the protein present. Essentially, it is a measure of the extent to which a protein undergoes chlorination at a particular site. The extent of chlorination can vary widely depending on the physiochemical properties of the specific sequence in the protein, similar to trends observed with phosphorylation conditions [36]. These properties might include the reactivity of the residue, its size, charge, hydrophobicity, and its accessibility in the protein structure. Some residues might have characteristics that make them more susceptible to chlorination, such as a high reactivity or a particular spatial orientation that exposes them to the chlorinating agent. On the other hand, some residues might be more resistant to modification due to low reactivity, steric hindrance, or because they are buried within the protein structure and not easily accessible. Therefore, understanding the chlorination stoichiometry can provide important insights into the functional consequences of protein chlorination, and how it may affect the protein stability, conformation, interaction with other molecules, and ultimately its biological function.

Our findings reveal that the proteins chlorinated under SAEW exposure are predominantly related to chaperones and translation processes (Figure S7). Notably, chaperone proteins such as DnaK (Hsp70) and chaperonin GroEL (Hsp60) are ATP-dependent molecular chaperones that mediate protein folding and protect proteins from oxidative stress-induced unfolding and aggregation [37,38]. Previous research has highlighted that protein synthesis, particularly heat shock proteins like DnaK and GroEL, may shut down when cells are incubated with sodium hypochlorite (NaOCl) [39]. Moreover, these heat shock proteins were observed to have reduced relative intensity in two-dimensional polyacrylamide gel electrophoresis compared to control cells. On the other hand, the presence of ATP enables DnaK to inhibit protein misfolding and/or aggregation and promote refolding. However, when cells are exposed to HOCl, this refolding may cause a sudden drop in cellular ATP levels, leading to the inhibition of DnaK [38]. Our results showed a down-regulation in chaperone-related proteins (DnaK, DnaJ, GroES) (Table S2). Furthermore, chlorinated DnaK was highly expressed compared to the control group (Table 2). Previous research has reported that DnaK and GroEL were key targets of HOCl modification [38], and our findings corroborate this, further illustrating that these proteins are heavily chlorinated under SAEW conditions (Table 2).

ClpB is part of the AAA+ family (ATPases associated with diverse cellular activities) and, in conjunction with DnaK, forms the DnaK-ClpB bi-chaperone system, which is capable of disaggregating stress-denatured proteins [40]. Nutrient deprivation can lead to protein aggregation due to the reduction of ATP, prompting the transition into the VBNC state [36]. Consequently, an increase in ATP can stimulate the DnaK-ClpB bi-chaperone system, facilitating the disaggregation of proteins [41]. Once the proteins are disaggregated, VBNC cells are capable of resuscitation and regrowth. Our study found that DnaK and ClpB proteins were down-regulated (Table S2). The suppression of these proteins might drive the cells towards the VBNC state, inhibiting the formation of the DnaK-ClpB bi-chaperone system and, subsequently, the resuscitation of the cells.

Elongation factor Tu (EF-Tu) constitutes up to 6–10% of the total protein expression in bacteria such as *E. coli* and *Mycoplasma pneumoniae*, making it one of the most abundant proteins in these organisms [42]. As previously mentioned, EF-Tu is essential for the translation process, binding to aminoacyl-tRNA and transporting it to the A site of the ribosome. Moreover, recent studies have discovered additional roles for EF-Tu, which include binding to immune system regulators to increase virulence and promote immune system evasion, adhering to fibronectin or other components of the extracellular matrix (ECM) to aid in invasion or adhesion into host cells, and associating with MreB to stimulate the production of MreB filaments that regulate cell shape [43]. These secondary roles are commonly referred to as “moonlighting” functions. Given that EF-Tu is one of the most abundant proteins and participates in many physiological functions in bacteria, its regulation is critical [43]. Our results identified six highly expressed chlorinated sites in this protein (Table 2), suggesting that SAEW might modulate the activation or inactivation of both translational and moonlighting functions through EF-Tu chlorination.

## 4. Materials and Methods

### 4.1. Bacterial Cultures and Preparations

*Listeria monocytogenes* BCRC14845 was purchased from the Bioresource Collection and Research Center (BCRC), Food Industry Research and Development Institute (FIRDI), Taiwan. The fresh stain was recovered on brain heart infusion broth (BHIB) and incubated at 37 °C for 24 h. The initial population of bacteria was determined to be approximately 8 log colony-forming units (CFU)/mL.

### 4.2. Preparation of Slightly Acidic Electrolyzed Water (SAEW)

SAEW was generated using a +HOCl water generator system (+HOCl Co., Taoyuan City, Taiwan). The final pH and oxidation-reduction potential values were measured with a dual-channel CyberScan pH meter (Eutech Instrument pH510, Singapore). The available chlorine concentration (ACC) was determined using a free chlorine portable photometer (HI-96701; Hanna instrument, Woonsocket, RI, USA). The SAEW were produced and diluted with deionized (DI) water to obtain the desired ACCs of 4, 6, 8, 10, and 20 mg/L ranging in pH 5.5–6.0.

### 4.3. Sample Treatment

The *L. monocytogenes* culture was enriched with BHIB at 37 °C for 18–20 h before further use. Several treatments were performed by mixing four microliters of pathogens into six microliters of SAEW, mixed thoroughly using a vortex, and allowed to incubate for 5 min. After 5 min reaction, 0.5 mL of 0.5% sodium thiosulfate (Na_2_S_2_O_3_) was added to the reaction mixture to quench the reaction of the remaining free chlorine.

### 4.4. Flow Cytometry Analysis of Treated Samples

The LIVE/DEAD^TM^ BacLight^TM^ bacterial viability kit (L7012) containing SYTO^®^ 9 and propidium iodide (PI) dyes were used to determine the viable and dead cells, respectively. Flow cytometry analysis was carried out on an Attune NxT Acoustic Focusing cytometer (Thermo Fisher Scientific, Rockford, IL, USA). The staining procedure was followed using the manufacturer’s instructions. The stained untreated pathogens were served as a control for the determination of viable cells, and heat treated cells (100 °C for 15 min) were used to represent dead cells. The stained controls were run, and a quadrant gate was used to indicate the viable and dead cell regions.

### 4.5. Scanning Electron Microscopy (SEM) Analysis

SEM analysis was conducted based on the method [21]. Briefly, *L. monocytogenes* was treated with SAEW in different ACCs, centrifuged at 2350× *g* for 10 min, and washed three times with 0.2 M sterile phosphate buffer saline (PBS, pH 7.4). After fixation and dehydration through a graded ethanol series (30, 50, 70, 80, and 90%) it was then placed in 55 °C for 1 min before finally being stored at −20 °C for future use. Before SEM analysis, the samples were coated with platinum for 30 s in an ion sputter (E-1010, Hitachi, Tokyo, Japan) and then examined in a scanning electron microscope (FESEM S-4800, Hitachi, Tokyo, Japan).

### 4.6. Whole-Protein Extraction for Proteomic Analysis

The treated bacterial pellets were re-suspended in a lysis buffer (12 mM sodium deoxycholate, 12 mM sodium N-lauroylsarcosinate, 10 mM Tris-HCl; pH 9.0, and 1X Protease and Phosphate Inhibitor Cocktail), extracted with ultrasonic disruption for 2 min in an ice bath. The mixture was then mixed with 1 mm ceramic beads and 0.1 mm glass beads. The cells were lysed with a cell homogenizer (FastPrep-24; MP Biomedicals, LLC, Santa Ana, CA, USA) for 60 s in an ice bath, according to our previous studies [9,10]. After centrifugation, the supernatant was collected as whole-protein extract and stored at −80 °C for further use.

### 4.7. Mass Spectrometric Analysis

#### 4.7.1. In Solution Digestion

The samples were reduced with 10 mM dithiothreitol and then alkylated using 50 mM iodoacetamide at 37 °C for 30 min. In-solution digestion was conducted based on our previous study [9] then desalted by using StageTips packing with an Empore SDB-XC disk (3M, St. Paul, MN, USA). The proteolytic peptides were quantified using Pierce Quantitative Colorimetric Peptide Assay (Thermo Scientific, Rockford, IL, USA) according to the manufacturer’s instructions.

#### 4.7.2. Tandem Mass Tags (TMT) Labeling

The dried peptide samples were re-suspended in 200 mM HEPES (pH 8.5), and processed according to the manufacturer’s instruction. Briefly, each TMT label was added to each peptide sample and incubated for 1 h at room temperature. After TMT labeling, 2% of hydroxylamine was added to each sample and incubated for 15 min to quench the reaction.

#### 4.7.3. SDB Fractionation and Desalted

All TMT-labeled samples were acidified and combined prior to being desalted and basic reverse-phase fractionated using SDB-XC StageTips. The peptide sample was loaded into the StageTips, desalted with 0.1% TFA and water, and eluted with a sequential gradient of 2–32% of ACN plus 5 mM ammonium hydroxide. In total, 16 fractions were collected and pooled in the following sequence: Fraction 1 with fraction 9, fraction 2 with fraction 10, fraction 3 with fraction 11, etc. All eight pooled peptides were then dried with a vacuum evaporator and re-suspended in 0.1% TFA and desalted prior to LC-MS/MS analysis.

#### 4.7.4. LC-MS/MS

Peptide samples were separated on a 75 μm × 250 mm Thermo Scientific Acclaim PepMap100 C18 Nano LC column and eluted with a linear gradient from 5% to 35% of solvent B (ACN with 0.1% formic acid) in 90 min with a flow rate of 300 nL/min. The positive ion mode with a nano-electrospray ionization (ESI) source was used. The mass scan range for the Orbitrap system was *m*/*z* 400 to 1600, with a maximum injection time of 50 ms, and detection at a mass resolution of 120 K in the Orbitrap Fusion^TM^ Tribrid^TM^ mass spectrometer (Thermo Scientific, Rockford, IL, USA).

#### 4.7.5. Data Analysis and Protein Network Construction

All MS/MS data were analyzed using MaxQuant software (v 1.6.6.0; Max Planck Institute of Biochemistry, Martinsried, Germany) for peptide identification, protein inference, and TMT reporter quantification. Peptides were identified with a false discovery rate (FDR) of <0.01 when applying a decoy database search. Protein sequence information of *L. monocytogenes* serovar 1/2a was obtained from UniProtKB (downloaded on 17 January 2022). All MS/MS spectra were searched against the database with variable modifications of the oxidation (M), oxidation of cysteine (+32 or +48), chlorination (+34), and dichlorination (+68) of tyrosine or tryptophan residues and fixed modification set as carbamidomethyl (C). The maximum missed cleavages of trypsin were 2.

### 4.8. Statistical Analysis

All the statistical analysis was performed using one-way ANOVA with GraphPad Prism (v 5.0; GraphPad, San Diego, CA, USA). Tukey’s multiple comparison test was used for multiple comparisons of means with a significance level of 0.05. An adjusted *p*-value of <0.05 was set as the threshold in the GO and STRING annotation.

## 5. Conclusions

This study has revealed that *Listeria monocytogenes* can enter a viable but nonculturable (VBNC) state when subjected to slightly acidic electrolyzed water (SAEW) treatment at concentrations of 8–10 mg/L. Despite cell shrinkage, the cell membrane integrity remains similar to the control group. Through quantitative proteomics, we identified 203 differentially expressed proteins (DEPs), which included 78 up-regulated and 125 down-regulated proteins. Functional enrichment analyses, including Gene Ontology (GO) and Kyoto Encyclopedia of Genes and Genomes (KEGG), revealed that these significant DEPs were primarily involved in the processes of ribosome, biosynthesis of secondary metabolites, and aminoacyl-tRNA biosynthesis. Interestingly, although SAEW treatment seemed to stimulate the initial steps of protein synthesis, a global down-regulation of the translation process was inferred, likely due to the shortage of elongation factors, aminoacyl-tRNA synthetases (AARSs), and other translation-related proteins. Importantly, we identified 31 chlorinated peptides from 22 chlorinated proteins, demonstrating that SAEW-induced protein chlorination indeed occurs, with prominent targets including the elongation factor Tu and chaperone proteins. These chlorinated proteins followed a conserved sequence motif, Rxxxxx[cY], and were involved in crucial biological processes such as chaperone cofactor-dependent protein refolding, translation, and organophosphate metabolic processes. Therefore, our findings suggest that SAEW triggers alterations in the protein translation process, and promotes a compensatory biosynthesis of ribosomes, but an imbalance in elongation factors, and AARSs levels impede recovery, thus leading to the VBNC state.

## Figures and Tables

**Table 1:** Recovery of culturable *L. monocytogenes* (BCRC14845) cells after 5-min treatment of different concentrations of chlorine in slightly acidic electrolyzed water (SAEW) and recovered on brain heart infusion agar (BHIA) for 24 h.

Chlorine Concentration (mg/L)	Recovery on BHIA (log CFU/mL)	Reduction on BHIA (log CFU/mL)
Control	8.26 ± 0.09	-
4	7.95 ± 0.17	0.31
6	7.54 ± 0.44	0.72
8	ND	8.26
10	ND	8.26
20	ND	8.26

Slightly acidic electrolyzed water: pH = 5.66 ± 0.08, ORP = 855.42 ± 89.79 Mv; ND: no detectable colonies observed to grow on plates (detection limit of 1 log CFU/mL).

**Table 2:** The list of quantifiable chlorinated peptides of *L. monocytogenes* after treatment with SAEW.

Protein IDs	Identified Sequence	Gene Symbol Cl Residue	Fold Change (log_2_)	
			SA20_CTL	SA10_CTL
Q8Y5V1	EFVPLLV**Y**SPR	deoB, Y350	1.038	1.102
P0DJM2	IINEPTAAALA**Y**GMDK	dnaK, Y153	1.180	1.155
Q8Y8G1	MDDL**Y**SEFGEQMDEVAER	dps, Y50	0.884	0.590
P64074	TSEEMVT**W**YEEMITK	eno, W273	1.809	0.564
P64074	TSEEMVTW**Y**EEMITK	eno, Y274	1.500	1.024
Q8Y7G7	LSVDY**Y**GAATPVNQMASISVPEAR	frr, Y45	0.869	1.217
Q8Y421	GV**Y**TMQFDHYEEVPK	fusA, Y666	2.352	2.508
Q8Y6D3	INDLGLPA**Y**DAMVLTLTK	gatB, Y313	1.455	1.589
Q8Y571	VIPY**W**MDTIAPEIK	gpmA, W162	1.282	1.615
B8DH59	HEAVGVGFNAANGE**W**INMIDAGIVDPTK	groEL, W482	1.543	1.041
B8DH59	GYTSP**Y**MVTDSDK	groEL, Y201	1.044	1.056
A0A0H3GEZ8	**Y**GLIETN	hpf, Y181	0.882	0.951
A0A0H3GEZ8	GENIEVTEPIRD**Y**VEK	hpf, Y20	1.310	0.927
C1KV98	**W**DVADLMD**Y**IK	ispH, W175; Y183	0.334	0.587
Q8YAE0	LAEK**Y**GVSIR	lmo0191, Y143	−0.053	0.076
Q8Y8D7	K**Y**MITSK	nadK1, Y3	0.262	0.292
Q8Y6C8	VTAEG**Y**VILNK	pcrB, Y112	2.267	2.457
Q71XR3	NFGAP**Y**ELIK	pdxS, Y194	1.158	1.349
P58641	ANDPVAA**Y**NQVLKE**W**NA	pyrF, Y224; W231	−0.504	−0.326
Q927L9	EQLIFPEID**Y**DQVSK	rplE, Y143	−0.272	−0.532
Q8Y6T6	RP**Y**APGQHGPTQR	rpsD, Y31	1.469	0.732
Q8Y6T6	HM**Y**GLTER	rpsD, Y61	1.781	1.652
Q71WF0	HVPV**Y**VQEDMVGHK	rpsS, Y61	0.790	1.068
Q8Y6U0	**Y**SGQVQMHIVPFTEIQEVIK	thiI, Y233	0.446	0.549
Q8Y6M7	TEMPNGQTVQD**Y**ITEAITK	tsf, Y125	1.771	1.791
Q8Y422	QVGVP**Y**IVVFMNK	tuf, Y130	3.229	2.687
Q8Y422	DLLTE**Y**EFPGDDIPVIK	tuf, Y161	1.010	0.921
Q8Y422	IDELMEAVDS**Y**IPTPER	tuf, Y199	1.353	1.216
Q8Y422	LLD**Y**AEAGDNIGALLR	tuf, Y269	0.157	0.229
Q8Y422	G**Y**ADAQAYDQIDGAPEER	tuf, Y41	1.229	1.230
Q8Y422	HYAHVDCPGHAD**Y**VK	tuf, Y88	0.538	1.220

## Supplementary Materials

The following supporting information can be downloaded at: https://www.mdpi.com/article/10.3390/ijms241310616/s1.
