# Supplementary material for: Quantitative Proteomic Analysis on the Slightly Acidic Electrolyzed Water Triggered Viable but Non-Culturable Listeria monocytogenes
Source: Int J Mol Sci. 2023 Jun 25;24(13):10616. doi: 10.3390/ijms241310616 (PMC10341410; doi:10.3390/ijms241310616)
Supplement: Supplementary file 1 [file ijms-24-10616-s001.zip › ijms-2394566-supplementary.pdf]

Table S1. The up-regulated proteins of *L. monocytogenes* after treatment with slightly acidic electrolyzed water in different chlorine concentrations. Note: SA20, 20 mg/L of SAEW; SA10, 10 mg/L of SAEW; C, control. The mark "#" is fold change (log_2) > 1, $p < 0.05$.

Protein IDs	Name (recommend name)	Entry Name	Gene name	Locus	Fold change (log_2)	
					SA20_CTL	SA10_CTL
P66401	30S ribosomal protein S14 type Z	RS14Z_LISMO	rpsZ	lmo2619	2.977#	2.746#
P0A491	50S ribosomal protein L35	RL35_LISMO	rpmI	lmo1784	2.291#	2.187#
P67288	UPF0154 protein lmo1306	Y1306_LISMO	lmo1306	lmo1306	1.804#	1.702#
Q7AP57	Glycine betaine transporter BetL	Q7AP57_LISMO	betL	lmo2092	1.797#	1.671#
Q8Y491	Probable tautomerase lmo2564	Y2564_LISMO	lmo2564	lmo2564	1.652#	1.801#
Q8Y443	50S ribosomal protein L24	RL24_LISMO	rplX	lmo2621	1.528#	1.300#
Q927M5	50S ribosomal protein L30	RL30_LISMO	rpmD	lmo2614	1.339#	1.314#
P66103	50S ribosomal protein L20	RL20_LISMO	rplT	lmo1783	1.311#	1.153#
Q8Y447	50S ribosomal protein L15	RL15_LISMO	rplO	lmo2613	1.041#	1.019#
Q8Y3I1	Ribonuclease P protein component	RNPA_LISMO	rnpA	lmo2855	1.033#	1.331#
P0A4Q8	UPF0145 protein lmo0208	Y208_LISMO	lmo0208	lmo0208	0.974	1.098#
Q8Y853	UPF0637 protein lmo1065	Y1065_LISMO	lmo1065	lmo1065	0.953	0.891
Q8Y9C1	FMN-dependent NADH:quinone oxidoreductase 1	AZOR1_LISMO	azoR1	lmo0611	0.919	0.776
Q8Y6C6	Phosphoribosylamine--glycine ligase	PUR2_LISMO	purD	lmo1764	0.913	0.967
Q8YAB4	2-C-methyl-D-erythritol 2,4-cyclodiphosphate synthase	ISPF_LISMO	ispF	lmo0236	0.857	0.689

Table S1. continued.

Protein IDs	Name (recommend name)	Entry Name	Gene name	Locus	Fold change (log_2)	
					SA20_CTL	SA10_CTL
Q92BL2	UPF0473 protein lmo1501	Y1501_LISMO	lmo1501	lmo1501	0.832	0.648
Q8Y5E4	Diadenylate cyclase	DACA_LISMO	dacA	lmo2120	0.770	0.800
P60426	50S ribosomal protein L2	RL2_LISMO	rplB	lmo2629	0.734	0.608
P66484	30S ribosomal protein S19	RS19_LISMO	rpsS	lmo2628	0.725	0.600
Q8Y556	3'-5' exoribonuclease YhaM	YHAM_LISMO	yhaM	lmo2220	0.717	0.672
Q927L9	50S ribosomal protein L5	RL5_LISMO	rplE	lmo2620	0.717	0.696
Q8Y458	50S ribosomal protein L13	RL13_LISMO	rplM	lmo2597	0.704	0.575
Q8YAR8	Single-stranded DNA-binding protein 1	SSB1_LISMO	ssb1	lmo0045	0.687	0.706
P66860	SsrA-binding protein	SSRP_LISMO	smpB	lmo2448	0.686	0.553
Q8Y753	Glycine--tRNA ligase alpha subunit	SYGA_LISMO	glyQ	lmo1459	0.655	0.563
Q8Y723	Putative pre-16S rRNA nuclease	YQGF_LISMO	lmo1502	lmo1502	0.649	0.635
P64032	Elongation factor P	EFP_LISMO	efp	lmo1355	0.649	0.594
P63439	Acyl carrier protein	ACP_LISMO	acpP	lmo1806	0.643	0.593
Q8Y4G1	HPr kinase/phosphorylase	HPRK_LISMO	hprK	lmo2483	0.623	0.529
Q8Y450	50S ribosomal protein L17	RL17_LISMO	rplQ	lmo2605	0.603	0.550
Q8Y7C6	Transcription antitermination protein NusB	NUSB_LISMO	nusB	lmo1359	0.540	0.466

Table S1. continued.

Protein IDs	Name (recommend name)	Entry Name	Gene name	Locus	Fold change (log_2)	
					SA20_CTL	SA10_CTL
Q8Y5S8	Ribonuclease Z	RNZ_LISMO	rnz	lmo1977	0.537	0.401
Q8Y445	50S ribosomal protein L18	RL18_LISMO	rplR	lmo2616	0.520	0.376
P66330	30S ribosomal protein S10	RS10_LISMO	rpsJ	lmo2633	0.518	0.334
P28764	Superoxide dismutase [Mn]	SODM_LISMO	sodA	lmo1439	0.500	0.531
Q8Y454	Energy-coupling factor transporter ATP-binding protein EcfA1	ECFA1_LISMO	ecfA1	lmo2601	0.489	0.442
Q8Y866	Peptide deformylase	DEF_LISMO	def	lmo1051	0.489	0.458
P66352	30S ribosomal protein S11	RS11_LISMO	rpsK	lmo2607	0.480	0.308
Q8Y5X4	Nucleoside diphosphate kinase	NDK_LISMO	ndk	lmo1929	0.473	0.383
Q8Y4G3	Pyrophosphatase PpaX	PPAX_LISMO	ppaX	lmo2481	0.466	0.455
Q8YA46	Hydroxyethylthiazole kinase	THIM_LISMO	thiM	lmo0316	0.455	0.338
Q8Y788	DNA mismatch repair protein MutL	MUTL_LISMO	mutL	lmo1404	0.451	0.422
Q8YAC7	Bifunctional protein TilS/HprT	TILS_LISMO	tilS/hprT	lmo0219	0.448	0.363
P66383	30S ribosomal protein S13	RS13_LISMO	rpsM	lmo2608	0.421	0.420
Q8Y708	Histidine--tRNA ligase	SYH_LISMO	hisS	lmo1520	0.419	0.346
Q8Y8C0	Peptide chain release factor 3	RF3_LISMO	prfC	lmo0988	0.395	0.347

Table S1. continued.

Protein IDs	Name (recommend name)	Entry Name	Gene name	Locus	Fold change (log_2)	
					SA20_CTL	SA10_CTL
Q8Y6C0	Phosphoribosylformylglycinamidine synthase subunit PurQ	PURQ_LISMO	purQ	lmo1770	0.394	0.286
Q8Y5W6	Cytidylate kinase	KCY_LISMO	cmk	lmo1939	0.391	0.316
Q8Y6X2	Threonine--tRNA ligase	SYT_LISMO	thrS	lmo1559	0.371	0.389
Q8Y7K1	Methylenetetrahydrofolate--tRNA-(uracil-5-)-methyltransferase TrmF	TRMFO_LISMO	trmFO	lmo1276	0.324	0.417
P61055	50S ribosomal protein L4	RL4_LISMO	rplD	lmo2631	0.323	0.201
Q8Y6T6	30S ribosomal protein S4	RS4_LISMO	rpsD	lmo1596	0.321	0.254
Q9ZIM1	Uncharacterized protein Lmo0216	Y216_LISMO	lmo0216	lmo0216	0.314	0.252
Q8YAR2	50S ribosomal protein L9	RL9_LISMO	rplI	lmo0053	0.302	0.247
Q8Y8P1	D-alanine--D-alanine ligase	DDL_LISMO	ddl	lmo0855	0.300	0.251
Q8Y729	5'-methylthioadenosine/S-adenosylhomocysteine nucleosidase	MTNN_LISMO	mtnN	lmo1494	0.298	0.222
Q8Y3Y6	Thymidylate kinase	KTHY_LISMO	tmk	lmo2693	0.296	0.226
Q8Y8K6	Serine-protein kinase RsbW	RSBW_LISMO	rsbW	lmo0894	0.292	0.208
Q48762	Uncharacterized PIN and TRAM-domain containing protein Lmo0234	Y234_LISMO	lmo0234	lmo0234	0.288	0.231
Q8Y653	Manganese-binding lipoprotein MntA	MNTA_LISMO	mntA	lmo1847	0.268	0.220
Q8Y4D8	Peptide chain release factor 2	RF2_LISMO	prfB	lmo2509	0.257	0.245

Table S1. continued.

Protein IDs	Name (recommend name)	Entry Name	Gene name	Locus	Fold change (log_2)	
					SA20_CTL	SA10_CTL
Q8Y5Z7	Methylglyoxal synthase	MGSA_LISMO	mgsA	lmo1906	0.206	0.187
Q8YAE2	Ribosomal RNA small subunit methyltransferase A	RSMA_LISMO	rsmA	lmo0188	0.198	0.224
Q8Y5Y0	3-phosphoshikimate 1-carboxyvinyltransferase	AROA_LISMO	aroA	lmo1923	0.178	0.215
O69192	Aminopeptidase C	PEPC_LISMO	pepC	lmo2338	0.154	0.127
Q8Y4C1	ATP synthase subunit beta 2	ATPB2_LISMO	atpD2	lmo2529	0.093	0.072
Q8Y6Z3	GTPase Obg	OBG_LISMO	obg	lmo1537	0.086	0.148
P66054	50S ribosomal protein L11	RL11_LISMO	rplK	lmo0248	0.533	0.424
Q927L2	50S ribosomal protein L22	RL22_LISMO	rplV	lmo2627	0.510	0.262
Q8Y7R3	Cobyric acid synthase	COBQ_LISMO	cobQ	lmo1208	0.455	0.715
P52331	RNA polymerase sigma factor SigA	SIGA_LISMO	sigA	lmo1454	0.286	0.330
Q93Q56	Glutamate 5-kinase	PROB_LISMO	proB	lmo1260	0.234	0.202
Q8Y7A9	Membrane protein insertase YidC 1	YIDC1_LISMO	yidC1	lmo1379	0.202	0.145
P65110	Translation initiation factor IF-1	IF1_LISMO	infA	lmo2610	0.159	0.208
Q8Y496	Autolysin, amidase	Q8Y496_LISMO	ami	lmo2558	0.744	-
Q8Y614	Cell cycle protein GpsB	GPSB_LISMO	gpsB	lmo1888	0.110	-

Table S1. continued.

Protein IDs	Name (recommend name)	Entry Name	Gene name	Locus	Fold change (log_2)	
					SA20_CTL	SA10_CTL
P58412	Cell wall teichoic acid glycosylation protein GtcA	GTCA_LISMO	gtcA	lmo2549	-	0.429
Q8Y4I2	Phosphoglycerate kinase	PGK_LISMO	pgk	lmo2458	-	0.229

Table S2. The down-regulated proteins of *L. monocytogenes* after treatment with slightly acidic electrolyzed water in different chlorine concentrations. Note: SA20, 20 mg/L of SAEW; SA10, 10 mg/L of SAEW; CTL, control. The mark "#" is fold change (log_2) > -1, p < 0.05. The mark "*" is the significant changes in different treatments ($p < 0.05$). The mark "-" is $p > 0.05$.

Protein IDs	Name (recommend name)	Entry Name	Gene name	Locus	Fold change (log_2)	
					SA20_CTL	SA10_CTL
Q8Y6S5	UPF0354 protein lmo1608	Y1608_LISMO	lmo1608	lmo1608	-2.435#	-1.980#
Q92EP8	Flotillin-like protein FloA	FLOA_LISMO	floA	lmo0392	-2.204#	-1.793#
Q8Y6Q3	Anthranilate phosphoribosyltransferase	TRPD_LISMO	trpD	lmo1631	-1.814#	-1.309#
Q9EYW9	Glutamate decarboxylase beta	DCEB_LISMO	gadB	lmo2363	-1.029#	-0.830
Q8Y754	Glycine--tRNA ligase beta subunit	SYGB_LISMO	glyS	lmo1458	-1.015#	-1.126#
Q8Y3T8	Probable transaldolase 1	TAL1_LISMO	tal1	lmo2743	-0.897	-0.765
Q8Y8H5	Sortase A	SRTA_LISMO	srtA	lmo0929	-0.724	-0.822
Q8Y870	GTP 3',8-cyclase	MOAA_LISMO	moaA	lmo1047	-0.670	-0.706
Q8Y6Z2	Glycerol kinase	GLPK_LISMO	glpK	lmo1538	-0.651	-0.473
Q9XDA6	Zinc uptake system ATP-binding protein ZurA	ZURA_LISMO	zurA	lmo1447	-0.612	-0.640
P0A485	50S ribosomal protein L31 type B	RL31B_LISMO	rpmE2	lmo2548	-0.600	-0.558
Q8Y551	Fumarate hydratase class II	FUMC_LISMO	fumC	lmo2225	-0.596	-0.406
Q8Y6M6	30S ribosomal protein S2	RS2_LISMO	rpsB	lmo1658	-0.587	-0.443
Q8Y822	GMP synthase [glutamine-hydrolyzing]	GUAA_LISMO	guaA	lmo1096	-0.568	-0.512
Q8Y7T0	Cobyrinate a,c-diamide synthase	CBIA_LISMO	cbiA	lmo1191	-0.557	-0.308
Q8Y7A7	Acylphosphatase	ACYP_LISMO	acyP	lmo1381	-0.542	-0.466

Table S2. continued.

Protein IDs	Name (recommend name)	Entry Name	Gene name	Locus	Fold change (log_2)	
					SA20_CTL	SA10_CTL
Q8Y570	Chaperone protein ClpB	CLPB_LISMO	clpB	lmo2206	-0.538	-0.458
Q8Y4C0	ATP synthase subunit alpha 2	ATPA2_LISMO	atpA2	lmo2531	-0.526	-0.483
P0A442	Pyruvate formate-lyase-activating enzyme	PFLA_LISMO	pflA	lmo1407	-0.524	-0.398
Q8Y5Z6	4-hydroxy-tetrahydrodipicolinate reductase	DAPB_LISMO	dapB	lmo1907	-0.507	-0.435
Q8Y457	tRNA pseudouridine synthase A	TRUA_LISMO	truA	lmo2598	-0.500	-0.503
Q8Y5N8	Isoleucine--tRNA ligase	SYI_LISMO	ileS	lmo2019	-0.499	-0.434
Q8Y446	30S ribosomal protein S5	RS5_LISMO	rpsE	lmo2615	-0.472	-0.363
Q8Y5L9	UDP-N-acetylmuramoyl-L-alanyl-D-glutamate--2,6-diaminopimelate ligase	MURE_LISMO	murE	lmo2038	-0.455	-0.371
Q8Y640	Peptide methionine sulfoxide reductase MsrA	MSRA_LISMO	msrA	lmo1860	-0.451	-0.332
Q8Y510	ATP-dependent helicase/deoxyribonuclease subunit B	ADDB_LISMO	addB	lmo2268	-0.446	-0.374
Q92CZ4	DNA-directed RNA polymerase subunit epsilon	RPOY_LISMO	rpoY	lmo1028	-0.431	-0.379
Q8YAF2	Methionine--tRNA ligase	SYM_LISMO	metG	lmo0177	-0.424	-0.328
Q92EH3	Heme-degrading monooxygenase	HDOX_LISMO	isdG	lmo0484	-0.417	-0.469
Q8YAB8	Lysine--tRNA ligase	SYK_LISMO	lysS	lmo0228	-0.415	-0.348
Q8Y5M1	UDP-N-acetylmuramoylalanine--D-glutamate ligase	MURD_LISMO	murD	lmo2036	-0.411	-0.529
Q8Y6K3	5-methyltetrahydropteroyltriglutamate--homocysteine methyltransferase	METE_LISMO	metE	lmo1681	-0.407	-0.503

Table S2. continued.

Protein IDs	Name (recommend name)	Entry Name	Gene name	Locus	Fold change (log_2)	
					SA20_CTL	SA10_CTL
P0A2X5	Adenine phosphoribosyltransferase	APT_LISMO	apt	lmo1524	-0.402	-0.444
Q8Y511	ATP-dependent helicase/nuclease subunit A	ADDA_LISMO	addA	lmo2267	-0.401	-0.332
Q8YAA4	50S ribosomal protein L1	RL1_LISMO	rplA	lmo0249	-0.399	-0.412
Q8YAB3	Glutamate--tRNA ligase	SYE_LISMO	gltX	lmo0237	-0.382	-0.351
Q8Y6D0	DNA ligase	DNLJ_LISMO	ligA	lmo1758	-0.373	-0.316
Q8Y4H0	Probable cell division protein WhiA	WHIA_LISMO	whiA	lmo2472	-0.368	-0.310
Q8Y6X9	Valine--tRNA ligase	SYV_LISMO	valS	lmo1552	-0.363	-0.300
Q8Y757	Probable manganese-dependent inorganic pyrophosphatase	PPAC_LISMO	ppaC	lmo1448	-0.360	-0.349
Q8Y7C5	Bifunctional protein FolD	FOLD_LISMO	folD	lmo1360	-0.356	-0.348
O31149	Phosphoenolpyruvate-protein phosphotransferase	PT1_LISMO	ptsI	lmo1003	-0.347	-0.280
Q8Y722	Alanine--tRNA ligase	SYA_LISMO	alaS	lmo1504	-0.347	-0.315
Q8Y449	Adenylate kinase	KAD_LISMO	adk	lmo2611	-0.342	-0.359
Q9RLT9	DNA-directed RNA polymerase subunit beta	RPOB_LISMO	rpoB	lmo0258	-0.340	-0.272
Q8Y3U2	NAD-dependent protein deacetylase	NPD_LISMO	cobB	lmo2739	-0.336	-0.279
Q93Q55	Gamma-glutamyl phosphate reductase	PROA_LISMO	proA	lmo1259	-0.336	-0.286
Q8Y6U8	Thiol peroxidase	TPX_LISMO	tpx	lmo1583	-0.324	-0.275

Table S2. continued.

Protein IDs	Name (recommend name)	Entry Name	Gene name	Locus	Fold change (log_2)	
					SA20_CTL	SA10_CTL
Q8Y3X7	Recombination protein RecR	RECR_LISMO	recR	lmo2702	-0.320	-0.402
Q8Y6S8	UDP-N-acetylmuramate--L-alanine ligase	MURC_LISMO	murC	lmo1605	-0.317	-0.264
Q8Y6X3	Probable GTP-binding protein EngB	ENGB_LISMO	engB	lmo1558	-0.314	-0.273
Q8Y3M5	tRNA uridine 5-carboxymethylaminomethyl modification enzyme MnmG	MNMG_LISMO	mnmG	lmo2810	-0.312	-0.276
Q8Y6D2	Glutamyl-tRNA(Gln) amidotransferase subunit A	GATA_LISMO	gatA	lmo1755	-0.312	-0.276
Q8Y564	Uroporphyrinogen decarboxylase	DCUP_LISMO	hemE	lmo2212	-0.309	-0.364
P66166	50S ribosomal protein L29	RL29_LISMO	rpmC	lmo2624	-0.302	-0.297
Q8Y6Z5	Probable transcriptional regulatory protein lmo1535	Y1535_LISMO	lmo1535	lmo1535	-0.302	-0.320
Q8Y9N5	Shikimate dehydrogenase (NADP(+))	AROE_LISMO	aroE	lmo0490	-0.302	-0.215
Q8Y641	Peptide methionine sulfoxide reductase MsrB	MSRB_LISMO	msrB	lmo1859	-0.297	-0.333
P0DJO8	Motility gene repressor MogR	MOGR_LISMO	mogR	lmo0674	-0.293	-0.283
Q8Y8D4	D-alanine--D-alanyl carrier protein ligase	DLTA_LISMO	dltA	lmo0974	-0.291	-0.170
Q8YAW2	Chromosomal replication initiator protein DnaA	DNAA_LISMO	dnaA	lmo0001	-0.291	-0.210
Q8Y7G1	DNA polymerase III PolC-type	DPO3_LISMO	polC	lmo1320	-0.289	-0.226
P0DJO9	Ribosomal protein L11 methyltransferase	PRMA_LISMO	prmA	lmo1471	-0.287	-0.264
P66699	DNA-directed RNA polymerase subunit alpha	RPOA_LISMO	rpoA	lmo2606	-0.285	-0.232

Table S2. continued.

Protein IDs	Name (recommend name)	Entry Name	Gene name	Locus	Fold change (log_2)	
					SA20_CTL	SA10_CTL
Q8Y5E6	Phosphoglucosamine mutase	GLMM_LISMO	glmM	lmo2118	-0.285	-0.241
P0DJM1	Chaperone protein DnaJ	DNAJ_LISMO	dnaJ	lmo1472	-0.284	-0.244
P58668	4-hydroxy-3-methylbut-2-en-1-yl diphosphate synthase (flavodoxin)	ISPG_LISMO	ispG	lmo1441	-0.283	-0.228
Q8Y9J4	UPF0237 protein lmo0533	Y533_LISMO	lmo0533	lmo0533	-0.275	-0.357
Q8Y6D3	Aspartyl/glutamyl-tRNA(Asn/Gln) amidotransferase subunit B	GATB_LISMO	gatB	lmo1754	-0.275	-0.195
Q8Y577	Tryptophan--tRNA ligase	SYW_LISMO	trpS	lmo2198	-0.273	-0.256
Q8Y5V1	Phosphopentomutase	DEOB_LISMO	deoB	lmo1954	-0.260	-0.274
Q8Y915	Glutamine--fructose-6-phosphate aminotransferase [isomerizing]	GLMS_LISMO	glmS	lmo0727	-0.246	-0.142
Q8Y7I9	S-ribosylhomocysteine lyase	LUXS_LISMO	luxS	lmo1288	-0.244	-0.162
P47847	Protein translocase subunit SecA 1	SECA1_LISMO	secA1	lmo2510	-0.244	-0.203
Q8Y6W9	Transcriptional repressor NrdR	NRDR_LISMO	nrdR	lmo1562	-0.238	-0.214
P53434	GTP cyclohydrolase 1 type 2 homolog	GCH1L_LISMO	lmo1452	lmo1452	-0.238	-0.264
Q8Y441	50S ribosomal protein L23	RL23_LISMO	rplW	lmo2630	-0.237	-0.215
Q8Y7F2	Bifunctional riboflavin kinase/FMN adenylyltransferase	RIBCF_LISMO	ribCF	lmo1329	-0.236	-0.198
Q8Y7F1	Polyribonucleotide nucleotidyltransferase	PNP_LISMO	pnp	lmo1331	-0.232	-0.190
Q8Y7G2	Proline--tRNA ligase	SYP_LISMO	proS	lmo1319	-0.229	-0.184

Table S2. continued.

Protein IDs	Name (recommend name)	Entry Name	Gene name	Locus	Fold change (log_2)	
					SA20_CTL	SA10_CTL
Q8Y4H1	NADPH dehydrogenase	NAMA_LISMO	namA	lmo2471	-0.228	-0.210
Q8Y421	Elongation factor G	EFG_LISMO	fusA	lmo2654	-0.227	-0.183
Q8Y793	Putative competence-damage inducible protein	CINA_LISMO	cinA	lmo1397	-0.227	-0.245
Q8Y6Y4	Cell shape-determining protein MreC	MREC_LISMO	mreC	lmo1547	-0.226	-0.268
Q7AP68	OpuCD protein	Q7AP68_LISMO	opuCD	lmo1425	-0.221	-0.277
Q8Y4A7	Thymidine kinase	KITH_LISMO	tdk	lmo2544	-0.219	-0.215
Q8YAD4	Bifunctional protein GlmU	GLMU_LISMO	glmU	lmo0198	-0.212	-0.146
Q8Y7A3	DNA translocase FtsK	FTSK_LISMO	ftsK	lmo1386	-0.211	-0.140
Q9AGE7	Co-chaperonin GroES	CH10_LISMO	groES	lmo2069	-0.211	-0.281
Q8Y6B1	Peptidase T	PEPT_LISMO	pepT	lmo1780	-0.206	-0.152
Q8Y8A0	N-acetyldiaminopimelate deacetylase	DAPEL_LISMO	lmo1012	lmo1012	-0.201	-0.191
Q8Y676	Methionyl-tRNA formyltransferase	FMT_LISMO	fmt	lmo1823	-0.194	-0.178
Q8Y6V9	Acetyl-coenzyme A carboxylase carboxyl transferase subunit alpha	ACCA_LISMO	accA	lmo1572	-0.187	-0.198
Q8YAE0	Carbohydrate deacetylase	YDJC_LISMO	lmo0191	lmo0191	-0.185	-0.237
P0DJM2	Chaperone protein DnaK	DNAK_LISMO	dnaK	lmo1473	-0.183	-0.217
Q8Y7N6	Ribonuclease PH	RNPH_LISMO	rph	lmo1238	-0.183	-0.223
P0DJP3	Protein translocase subunit SecA 2	SECA2_LISMO	secA2	lmo0583	-0.182	-0.127

Table S2. continued.

Protein IDs	Name (recommend name)	Entry Name	Gene name	Locus	Fold change (log_2)	
					SA20_CTL	SA10_CTL
Q8Y5L7	Ribosomal RNA small subunit methyltransferase H	RSMH_LISMO	rsmH	lmo2041	-0.177	-0.167
Q8Y7P1	Endonuclease MutS2	MUTS2_LISMO	mutS2	lmo1232	-0.170	-0.167
Q8Y5T8	Uncharacterized protein Lmo1967	Y1967_LISMO	lmo1967	lmo1967	-0.167	-0.144
Q8Y7G3	Putative zinc metalloprotease Lmo1318	Y1318_LISMO	lmo1318	lmo1318	-0.165	-0.135
Q8Y755	DEAD-box ATP-dependent RNA helicase CshB	CSHB_LISMO	cshB	lmo1450	-0.164	-0.157
Q8Y7Q1	Phenylalanine--tRNA ligase beta subunit	SYFB_LISMO	pheT	lmo1222	-0.151	-0.107
Q8Y6X8	Glutamate-1-semialdehyde 2,1-aminomutase 1	GSA1_LISMO	hemL1	lmo1553	-0.144	-0.110
Q8YAR1	Adenylosuccinate synthetase	PURA_LISMO	purA	lmo0055	-0.138	-0.174
Q8Y756	Probable endonuclease 4	END4_LISMO	nfo	lmo1449	-0.135	-0.116
Q8Y493	Arginine--tRNA ligase	SYR_LISMO	argS	lmo2561	-0.134	-0.071
Q8Y664	Carbamoyl-phosphate synthase small chain	CARA_LISMO	carA	lmo1836	-0.131	-0.154
Q8Y8V6	FMN-dependent NADH:quinone oxidoreductase 2	AZOR2_LISMO	azoR2	lmo0786	-0.114	-0.129
Q8Y6D6	Uncharacterized RNA methyltransferase lmo1751	Y1751_LISMO	lmo1751	lmo1751	-0.111	-0.073
Q8Y3T4	Serine--tRNA ligase	SYS_LISMO	serS	lmo2747	-0.086	-0.093
Q8Y4B8	ATP synthase subunit b	ATPF_LISMO	atpF	lmo2533	-0.083	-0.117
Q8Y7C3	Exodeoxyribonuclease 7 small subunit	EX7S_LISMO	xseB	lmo1362	-0.546	-0.462*

Table S2. continued.

Protein IDs	Name (recommend name)	Entry Name	Gene name	Locus	Fold change (log_2)	
					SA20_CTL	SA10_CTL
Q8Y3S3	Macro domain-containing protein lmo2759	Y2759_LISMO	lmo2759	lmo2759	-0.425	-0.339*
Q8Y4R7	Glucose-6-phosphate isomerase	G6PI_LISMO	pgi	lmo2367	-0.310	-0.179*
Q8Y709	Aspartate--tRNA ligase	SYD_LISMO	aspS	lmo1519	-0.283	-0.223*
Q8Y4F5	UvrABC system protein B	UVRB_LISMO	uvrB	lmo2489	-0.249	-0.191*
P0DJP2	Ribonuclease Y	RNY_LISMO	rny	lmo1399	-0.231	-0.184*
Q8Y6T8	Septation ring formation regulator EzrA	EZRA_LISMO	ezrA	lmo1594	-0.215	-0.151*
Q8YA96	DNA-directed RNA polymerase subunit beta'	RPOC_LISMO	rpoC	lmo0259	-0.205	-0.149*
Q8Y6H7	UPF0435 protein lmo1707	Y1707_LISMO	lmo1707	lmo1707	-0.149	-0.236*
Q8Y789	DNA mismatch repair protein MutS	MUTS_LISMO	mutS	lmo1403	-0.143	-0.074*
Q8Y6K9	2-succinyl-5-enolpyruvyl-6-hydroxy-3-cyclohexene-1-carboxylate synthase	MEND_LISMO	menD	lmo1675	-0.129	-0.078*
Q8Y7H5	UPF0291 protein lmo1304	Y1304_LISMO	lmo1304	lmo1304	-0.186*	-
Q8Y6Q5	N-(5'-phosphoribosyl)anthranilate isomerase	TRPF_LISMO	trpF	lmo1629	-	-0.288*

Table S3. Functional enrichment analysis on KEGG pathway of the DEPs of *L. monocytogenes* after treatment with SAEW.

Functional characterization	KEGG pathway	Up-regulated	Down-regulated
**Metabolism**			
Global and overview maps	01100 Metabolic pathways	aroA, thiM, pgk[1], proB, ndk, tmk, cmk, mtnN, mgsA, ispF, lmo2564, ddl, purD, acpP, atpD2, cobQ, purQ	proA, adk, metG, cbiA, lmo1012, dapB, folD, murC, gatA, luxS, metE, acyP, murD, gatB, gltX, fmt, gadB, murE, menD, ribCF, tal1, hemE, purA, dltA, cobB, apt, fumC, guaA, glmM, trpD, pgi, carA, ispG, glmS, trpF[1], accA, moaA, tdk, deoB, atpF, cinA, glpK, hemL1, atpA2, glmU
	01110 Biosynthesis of secondary metabolites	aroA, pgk[1], proB, ndk, ispF, purD, acpP, purQ	proA, trpF[1], dapB, hemL1, metE, accA, tal1, menD, trpD, ispG, adk, lmo1012, gadB, isdG, hemE, ribCF, fumC, pgi
	01120 Microbial metabolism in diverse environments	pgk[1], mgsA, lmo2564	dapB, tal1, folD, acyP, gadB, fumC, pgi, hemL1, accA
	01200 Carbon metabolism	pgk[1]	tal1, folD, fumC, pgi, accA
	01212 Fatty acid metabolism	-	accA
	01230 Biosynthesis of amino acids	aroA, pgk[1], proB, mtnN	proA, dapB, metE, tal1, trpD, trpF[1], lmo1012, luxS
	01232 Nucleotide metabolism	ndk, tmk, cmk	apt, tdk, adk, purA, guaA
	01250 Biosynthesis of nucleotide sugars	-	glmS, pgi, glmM, glmU
	01240 Biosynthesis of cofactors	thiM, ndk, cobQ	adk, folD, hemE, hemL1, purA,, carA, cbiA, menD, moaA, gltX, ribCF
	01220 Degradation of aromatic compounds	lmo2564	-
Carbohydrate metabolism	00010 Glycolysis/ Gluconeogenesis	pgk[1]	pgi
	00020 Citrate cycle (TCA cycle)	-	fumC
	00030 Pentose phosphate pathway	-	tal1, pgi, deoB
	00500 Starch and sucrose metabolism	-	pgi

	00520 Amino sugar and nucleotide sugar metabolism	-	glmS, pgi, glmM, glmU
	00620 Pyruvate metabolism	-	acyP, fumC, accA
	00640 Propanoate metabolism	mgsA	accA
	00650 Butanoate metabolism	-	gadB
Energy metabolism	00190 Oxidative phosphorylation	atpD2, ppaX	atpF, atpA2, ppaC
	00195 Photosynthesis	atpD2	atpF, atpA2
	00720 Carbon fixation pathways in prokaryotes	-	folD, fumC, accA
	00710 Carbon fixation in photosynthetic organisms	pgk[1]	-
Lipid metabolism	00061 Fatty acid biosynthesis	-	accA
	00561 Glycerolipid metabolism	-	glpK
Nucleotide metabolism	00230 Purine metabolism	ndk, purD, purQ	apt, adk, deoB, purA, guaA
	00240 Pyrimidine metabolism	ngk, tmk, cmk	tdk, carA
Amino acid metabolism	00250 Alanine, aspartate and glutamate metabolism	purQ	glmS, gadB, purA, carA
	00270 Cysteine and methionine metabolism	mtnN	metE, luxS
	00300 Lysine biosynthesis	-	dapB, murE, lmo1012
	00330 Arginine and proline metabolism	proB	proA
	00400 Phenylalanine, tyrosine and tryptophan biosynthesis	aroA	trpD, trpF[1]
	00270 Cysteine and methionine metabolism	mtnN	-
Metabolism of other amino acids	00410 beta-Alanine metabolism	-	gadB
	00430 Taurine and hypotaurine metabolism	-	gadB
	00450 Selenocompound metabolism	-	metE, metG
	00470 D-Amino acid metabolism	ddl, purQ	murD, dltA
Glycan biosynthesis and metabolism	00541 O-Antigen nucleotide sugar biosynthesis	-	glmU
	00550 Peptidoglycan biosynthesis	ddl	murC, murD, murE

	00552 Teichoic acid biosynthesis	-	dtlA
Metabolism of cofactors and vitamins	00730 Thigpsne metabolism	thiM	adk
	00740 Riboflavin metabolism	thiM	ribCF
	00760 Nicotinate and nicotinamide metabolism	-	cinA, cobB
	00790 Folate biosynthesis	-	moaA
	00670 One carbon pool by folate	-	fmt, folD
	00860 Porphyrin metabolism	cobQ	hemE, hemL1, chiA, isdG
	00130 Ubiquinone and other terpenoid-quinone biosynthesis	-	menD
Metabolism of terpenoids and polyketides	00900 Terpenoid backbone biosynthesis	ispF	ispG
Biosynthesis of other secondary metabolites	00332 Carbapenem biosynthesis	proB	proA
	00261 Monobactam biosynthesis	-	dapB
	00998 Biosynthesis of various antibiotics	acpP	-
Xenobiotics biodegradation and metabolism	00627 Aminobenzoate degradation	-	acyP
	00983 Drug metabolism - other enzymes	ndk	tdk, guaA
	00362 Benzoate degradation	lmo2564	-
	00622 Xylene degradation	lmo2564	-
	00621 Dioxin degradation	lmo2564	-
**Genetic information processing**			
Transcription	03020 RNA polymerase	-	rpoA, rpoB, rpoC
Translation	03010 Ribosome	rplK, rplM, rplO, rplQ, rplR, rplB, rplT, rplV, rplX, rpmD, rpmI, rplD, rplE, rplI, rpsJ, rpsK, rpsM, rpsZ, rpsS, rpsD	rplA, rplW, rpmC, rpmE2, rpsB, rprE
	00970 Aminoacyl-tRNA biosynthesis	thrS, glyQ, hisS	fmt, trpS, ileS, alaS, valS, metG, serS, aspS, glyS, proS, argS, pheT, gatA, gatB, lysS, gltX

Folding, sorting and degradation	03060 Protein export	yidC1	secA2
	04122 Sulfur relay system	-	moaA
	03018 RNA degradation	-	pnp, dnaK, rny
Replication and repair	03030 DNA replication	ssb1	ligA, polC
	03410 Base excision repair	-	nfo, ligA
	03420 Nucleotide excision repair	-	ligA. uvrB
	03430 Mismatch repair	ssb1, mutL	ligA, mutS, xseB, polC, mutS2
	03440 Homologous recombination	ssb1	polC, recR
**Environmental Information Processing**			
Membrane transport	02010 ABC transporters	efcA1, mntA	opuCD, zurA
	02060 Phosphotransferase system (PTS)	-	ptsl
	03070 Bacterial secretion system	yidC1	secA2
Signal transduction	02020 Two-component system	-	dnaA, dltA
	04013 MAPK signaling pathway – fly	sodA	-
	04016 MAPK signaling pathway – plant	ndk	-
	04066 HIF-1 signaling pathway	pgk[1]	-
	04068 FoxO signaling pathway	sodA	-
**Cellular processes**			
Cell growth and death	04112 Cell cycle - Caulobacter	-	dnaA, lmo1318
Cellular community - prokaryotes	02024 Quorum sensing	yidC1	gadB, secA2, luxS
	05111 Biofilm formation - Vibrio cholerae	-	luxS
	02026 Biofilm formation - Escherichia coli	-	luxS
Transport and catabolism	04146 Peroxisome	sodA	-
Cell motility	02040 Flagellar assembly	sigA	-
**Organismal systems**			
Endocrine system	03320 PPAR signaling pathway	-	glpK
Nervous system	04727 GABAergic synapse	-	gadB
Aging	04211 Longevity regulating pathway	sodA	-
	04212 Longevity regulating pathway - worm	sodA	dnaK

	04213 Longevity regulating pathway - multiple species	sodA	clpB
Environmental adaption	04626 Plant-pathogen interaction	-	glpK
**Human diseases**			
Cancer: overview	05200 Pathways in cancer	-	fumC
	05208 Chemical carcinogenesis - reactive oxygen species	sodA	-
Cancer: specific types	05211 Renal cell carcinoma	-	fumC
Infectious disease: bacterial	05150 Staphylococcus aureus infection	-	dltA
	05152 Tuberculosis	-	dnaK
Neurodegenerative disease	05016 Huntington disease	sodA	-
Cardiovascular disease	05415 Diabetic cardiomyopathy	-	glmS
	05417 Lipid and atherosclerosis	sodA	-
Endocrine and metabolic disease	04940 Type I diabetes mellitus		gadB
	04931 Insulin resistance		glmS
	04934 Cushing syndrome		fumC
Drug resistance: antimicrobial	01502 Vancomycin resistance	ddl	-
	01503 Cationic antimicrobial peptide (CAMP) resistance	-	dltA

[1], upregulated by low dose SA treatment (SA10 group)

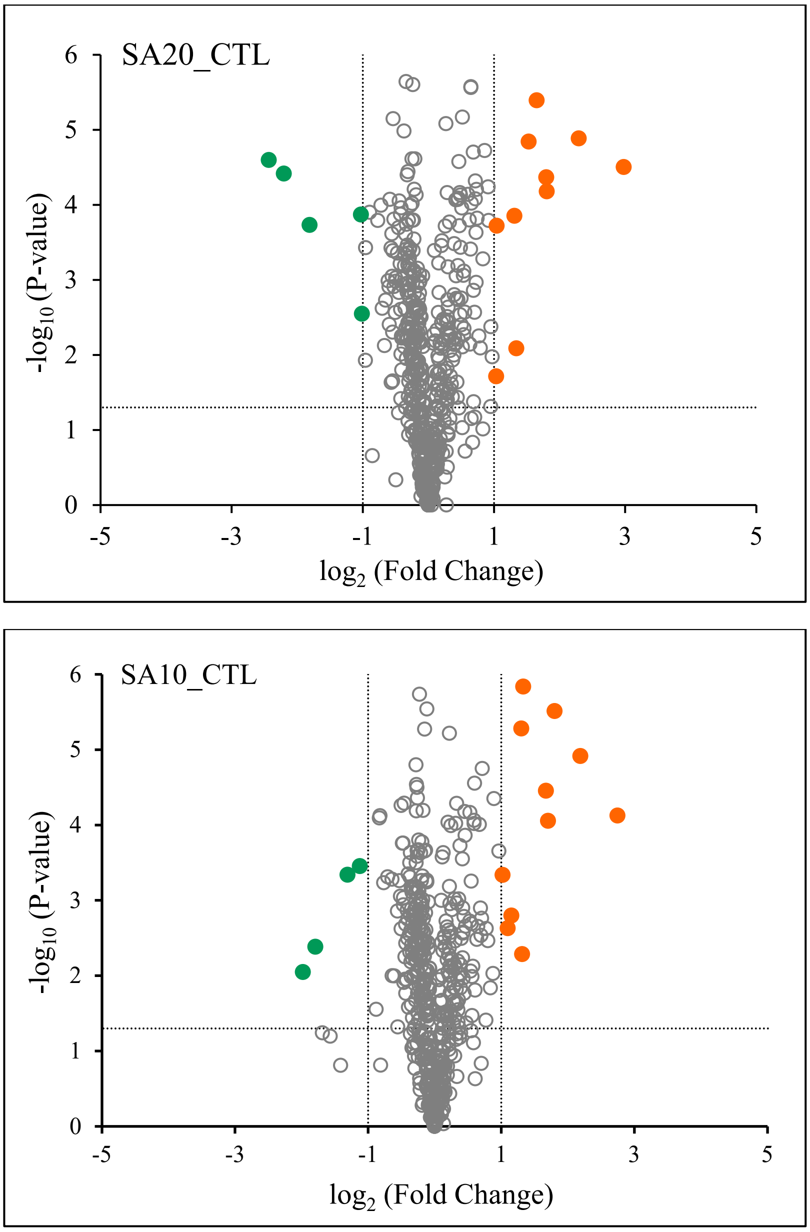

Figure S1. Volcano plot of significant differential expressed proteins (sDEPs) between various treatments of SAEW in *L. monocytogenes*. Note: SA20, 20 mg/L of ACC in SAEW; SA10, 10 mg/L of ACC in SAEW; CTL, control. The orange dots and green dots are significant up and down regulated, respectively. The dash line of x-axis on the left and right side respectively are Fold change = -2 and 2. The dash line in y-axis is P-value = 0.05.

tuf,Y130

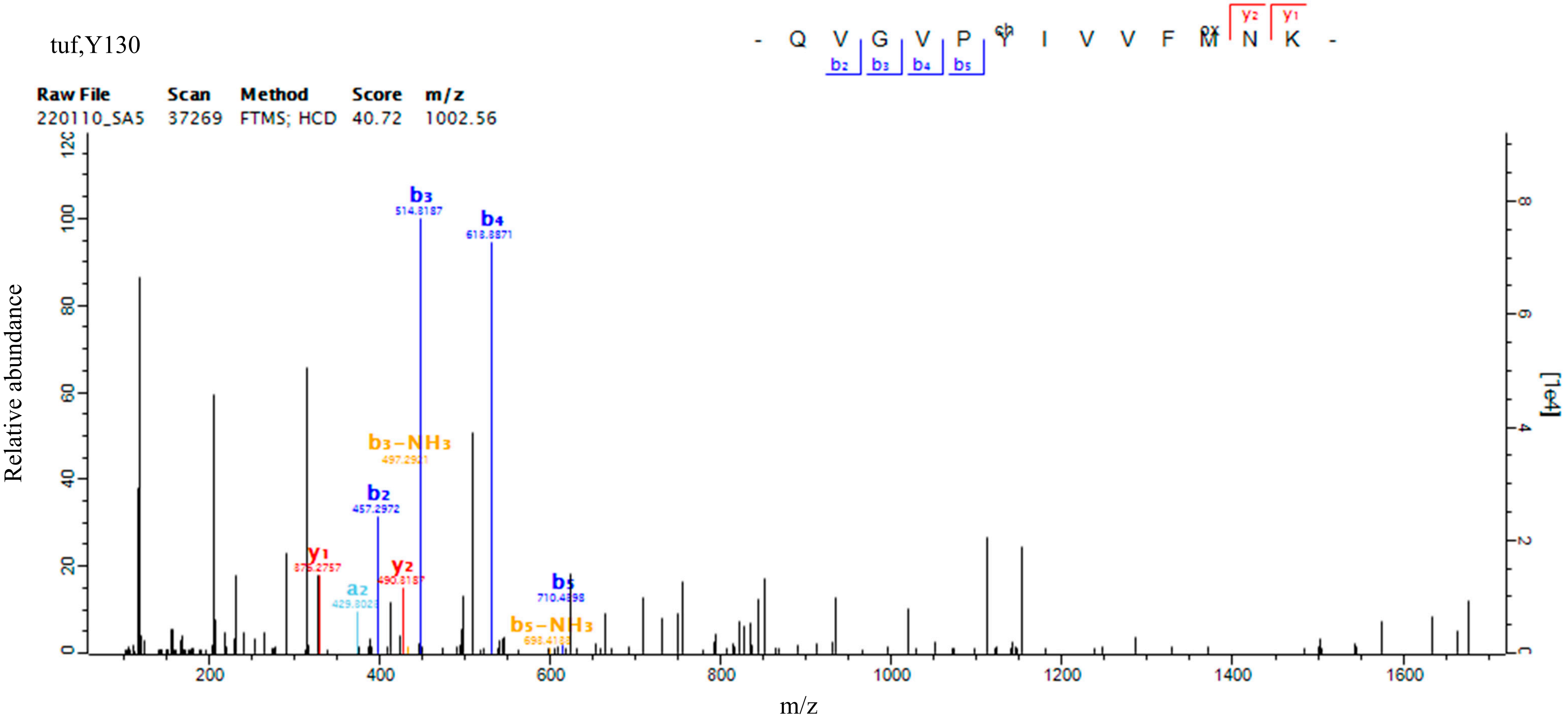

Figure S2. The high energy collision dissociation (HCD) mass spectrum of the chlorinated peptide QVGVP^{130}Y^{Cl}IVVFMNK at m/z 1002.56.

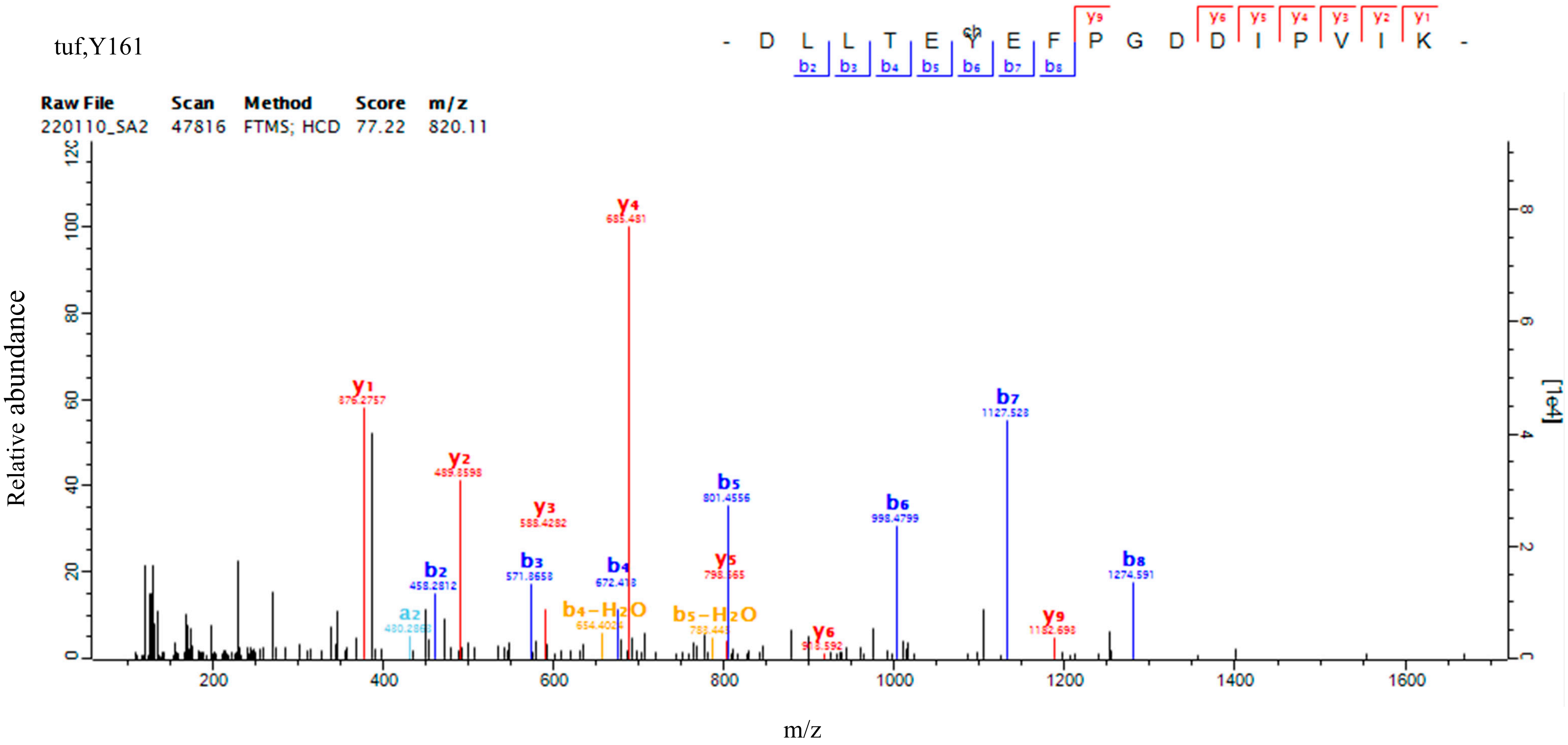

Figure S3. The high energy collision dissociation (HCD) mass spectrum of the chlorinated peptide DLLTE[161]Y^{Cl}EFPGDDIPVIK at m/z 820.11.

tuf,Y269

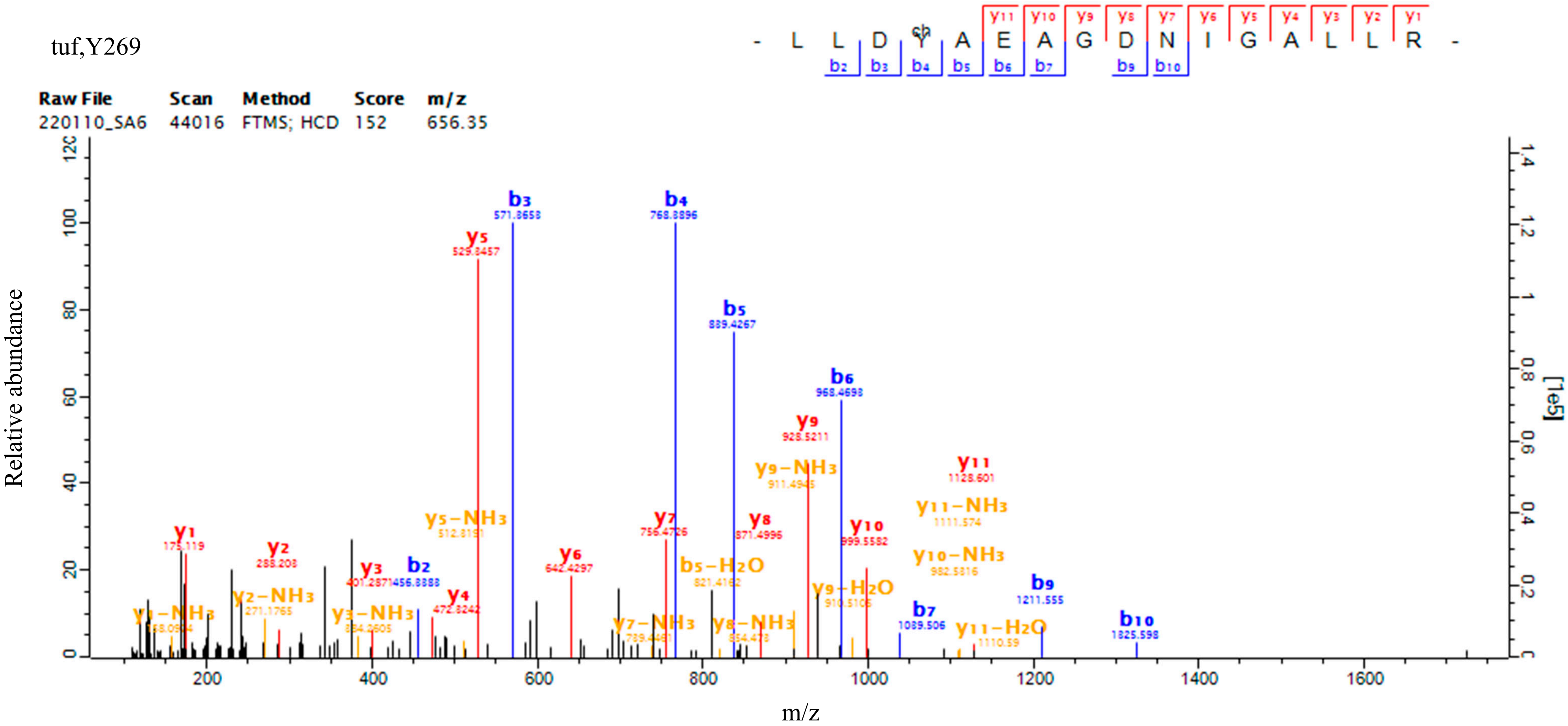

Figure S4. The high energy collision dissociation (HCD) mass spectrum of the chlorinated peptide LLD^{269}Y^{Cl}AEAGDNIGALLR at m/z 656.35.

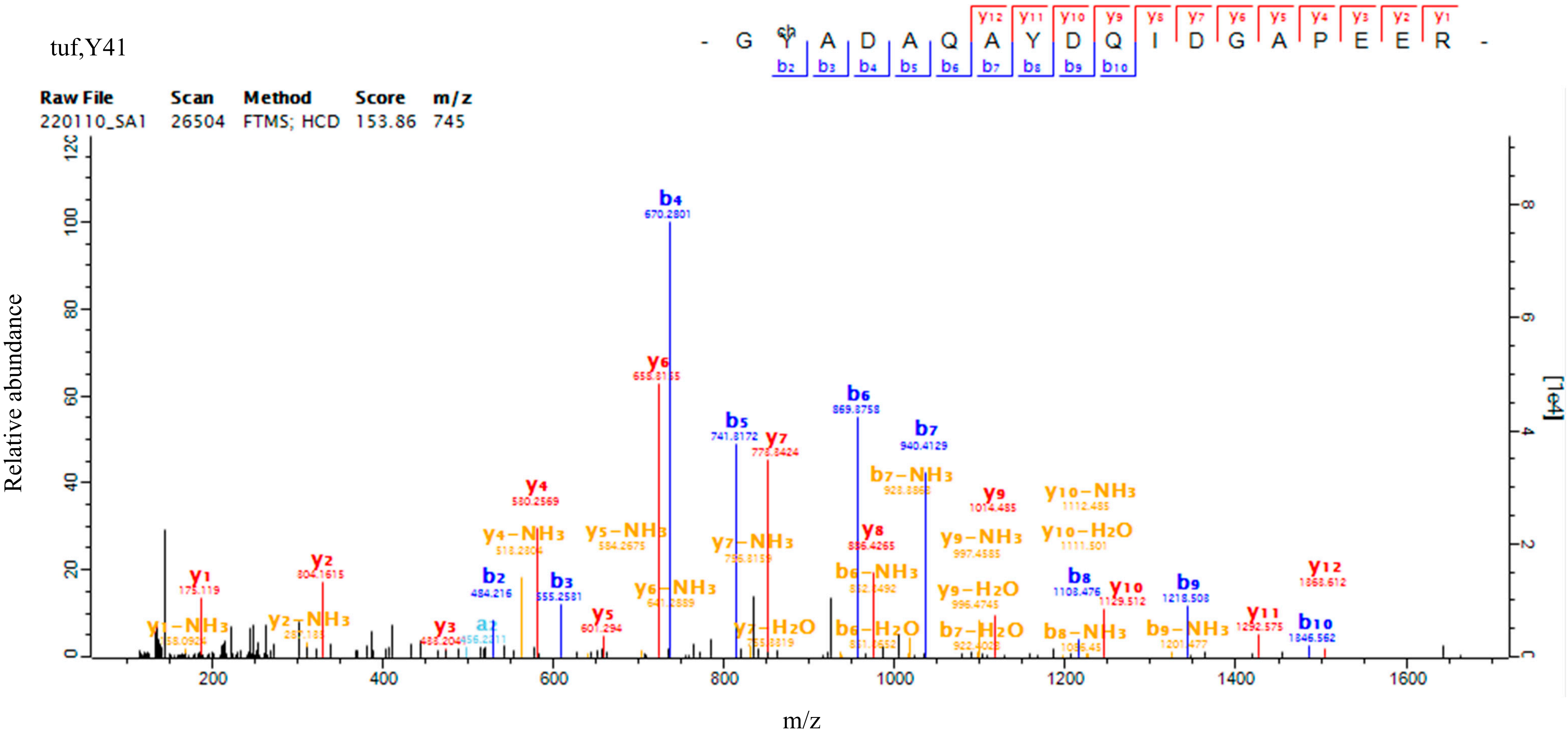

Figure S5. The high energy collision dissociation (HCD) mass spectrum of the chlorinated peptide G^{41}Y^{Cl}ADAQAYDQIDGAPEER at m/z 745.

tuf,Y88

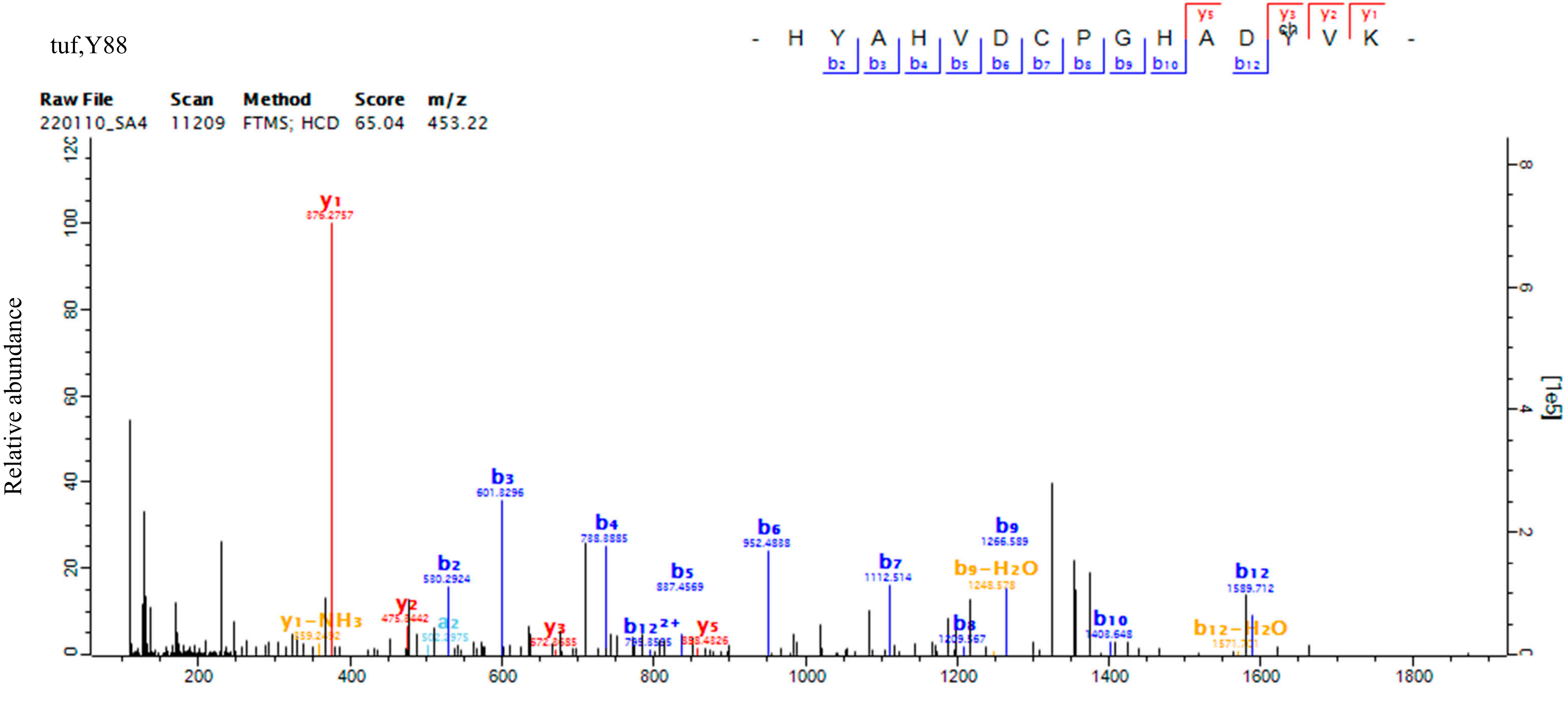

m/z

Figure S6. The high energy collision dissociation (HCD) mass spectrum of the chlorinated peptide HYAHVDCPGHAD^{88}Y^{Cl}VK at m/z 453.22.

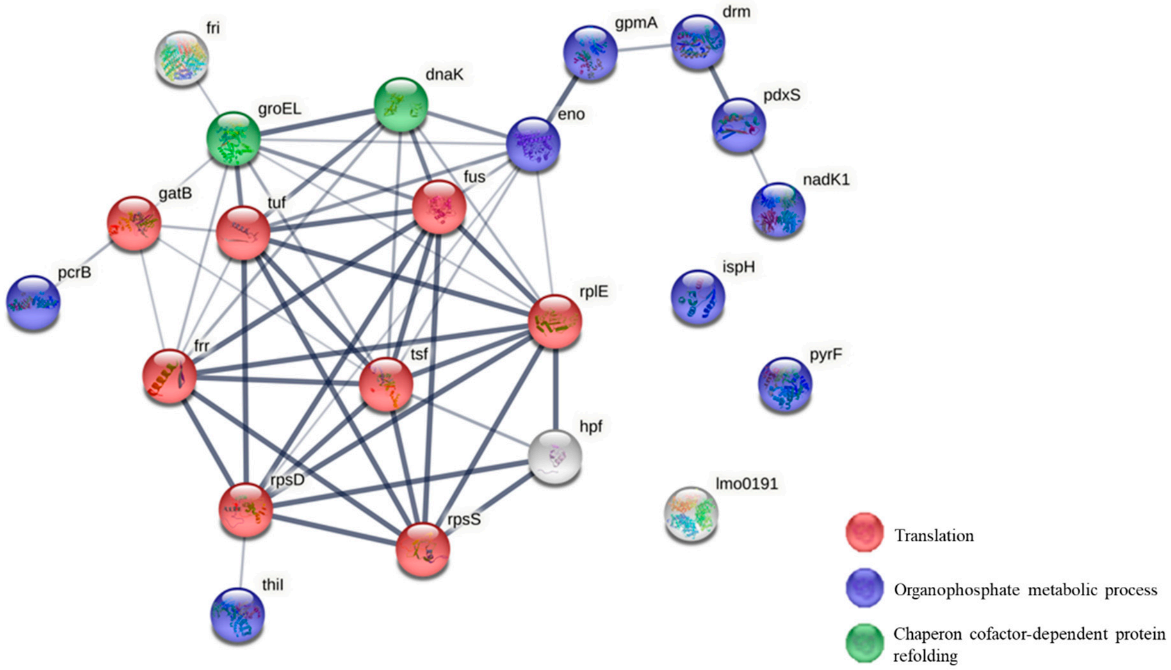

Figure S7. Protein-protein interaction of chlorination proteins in *L. monocytogenes*. Functional categorization was conducted based on Gene Ontology (GO) level and STRING Protein-Protein Interaction Networks. The average node degree is 3.88 and the average local clustering coefficient is 0.579.
